# Pharmacological pain management in patients with rheumatoid arthritis: a narrative literature review

**DOI:** 10.1186/s12916-025-03870-0

**Published:** 2025-01-29

**Authors:** Natasha Cox, Christian D. Mallen, Ian C. Scott

**Affiliations:** 1 Primary Care Centre Versus Arthritis, School of Medicine, Keele University, Keele, UK; 2 Haywood Academic Rheumatology Centre, Haywood Hospital, Midlands Partnership University NHS Foundation Trust, High Lane, Burslem, Staffordshire UK

**Keywords:** Rheumatoid arthritis, Pain, Analgesic, DMARD, Glucocorticoid

## Abstract

**Background:**

Pain is a major challenge for patients with rheumatoid arthritis (RA), with many people suffering chronic pain. Current RA management guidelines focus on assessing and reducing disease activity using disease-modifying anti-rheumatic drugs (DMARDs). Consequently, pain care is often suboptimal, with growing evidence that analgesics are widely prescribed to patients with RA, despite potential toxicities and limited evidence for efficacy. Our review provides an overview of pharmacological treatments for pain in patients with RA, summarising their efficacy and use.

**Findings:**

Thirteen systematic reviews of drug efficacy for pain in patients with RA were included in this review. These showed moderate- to high-quality evidence from clinical trials in more contemporary time-periods (mainly 1990s/2000s for synthetic DMARDs and post-2000 for biological/targeted synthetic DMARDs) that, in patients with active RA, short-term glucocorticoids and synthetic, biologic, and targeted synthetic DMARDs have efficacy at reducing pain intensity relative to placebo. In contrast, they showed low-quality evidence from trials in more historical time-periods (mainly in the 1960s–1990s for opioids and paracetamol) that (aside from naproxen) analgesics/neuromodulators provide any improvements in pain relative to placebo, and no supportive evidence for gabapentinoids, or long-term opioids. Despite this evidence base, 21 studies of analgesic prescribing in patients with RA consistently showed substantial and sustained prescribing of analgesics, particularly opioids, with approximately one quarter and > 40% of patients receiving chronic opioid prescriptions in each year in England and North America, respectively. Whilst NSAID prescribing had fallen over time across countries, gabapentinoid prescribing in England had risen from < 1% of patients in 2004 to approximately 10% in 2020. Prescribing levels varied substantially between individual clinicians and groups of patients.

**Conclusions:**

In patients with active RA, DMARDs have efficacy at reducing pain, supporting the role of treat-to-target strategies. Despite limited evidence that analgesics improve pain in patients with RA, these medicines are widely prescribed. The reasons for this are unclear. We consider that closing this evidence-to-practice gap requires qualitative research exploring the drivers of this practice, high-quality trials of analgesic efficacy in contemporary RA populations, alongside an increased focus on pain management (including pharmacological and non-pharmacological options) within RA guidelines.

**Supplementary Information:**

The online version contains supplementary material available at 10.1186/s12916-025-03870-0.

## Background

Rheumatoid arthritis (RA) is a common autoimmune-mediated condition, characterised by persistent synovial inflammation (particularly of the hand and feet small joints, although any synovial joint can be affected). Raised inflammatory markers and autoantibodies (rheumatoid factor and/or anti-cyclic citrullinated peptide antibodies) are common. Contemporary RA management focuses on treat-to-target strategies. These involve measuring disease activity regularly—often using the Disease Activity Score for 28-Joint Counts (DAS28), which represents a composite score combining information on swollen and tender joint counts, the patient global assessment of disease activity, and inflammatory markers—and increasing treatment with disease-modifying anti-rheumatic drugs (DMARDs) until the “target” of remission or low disease activity is achieved [1]. The high prevalence of RA (which affects approximately 0.8% of adults in England [2], and 17.6 million people globally [3], and is rising in both contexts) and far-reaching personal and economic impacts (including increased disability [4] and unemployment [5]) mean that optimising the care patients with RA receive is a clinical priority.

Despite key therapeutic advances in reducing synovitis and disease activity, pain remains a major challenge in RA. Surveys demonstrate that approximately two-thirds of patients have daily pain [6] with most rating pain to be the health area they most want improved [7], and longitudinal studies show that despite biologic therapies pain is often uncontrolled [8, 9] (with 79% of patients with RA receiving biologics in the British Society for Rheumatology Biologics Registry belonging to a “persistent pain” trajectory). Pain has detrimental impacts on the quality of life [10], function [11], mental health [12], and fatigue levels [13] of patients with RA. The mechanisms driving pain in RA are complex, and often involve multiple pain types and pathways [14], with fibromyalgia particularly common [15].

Contemporary RA guidelines focus on reducing disease activity using DMARDs [16–18], providing few/no pain-specific recommendations. To date, the only RA pain-specific guideline is from the European Alliance of Associations for Rheumatology (EULAR) [19]. Underpinned by an umbrella review of non-pharmacological treatments, it advocates biopsychosocial approaches involving reducing synovitis and non-drug care. In the absence of a pain-specific focus in most RA guidelines, there is increasing evidence that its management involves the substantial prescribing of analgesics (particularly opioids) [20, 21], despite known risks (including overdose, fractures, and myocardial infarction with opioids, upper gastrointestinal complications and cardiovascular events with NSAIDs [22–24]), emerging data about other potential harms (such as fractures with gabapentinoids [25]), and limited trial evidence for efficacy. Our narrative review summarises evidence for (a) the efficacy of pharmacological treatments for pain in RA and (b) how they are being prescribed/used, outlining potential future research directions to reduce evidence-to-practice gaps. It provides complementary but distinct information to recent RA therapeutic reviews describing the role of immunosuppressive medicines to reduce disease activity [26, 27].

## Methods

### Efficacy evidence

We searched Medline and EMBASE (using the Ovid Platform from inception until July 2024) alongside RA guidelines to identify systematic reviews evaluating the efficacy of the following drugs for pain in RA: analgesics (paracetamol/acetaminophen, opioids, NSAIDs); gabapentinoids, anti-depressants, cannabinoids, and other neuromodulators; and drugs for disease activity (corticosteroids, synthetic DMARDs, biologic DMARDs, targeted synthetic DMARDs). We considered corticosteroids/DMARDs because pain is integral to the concept of disease activity, being directly considered in two American College of Rheumatology-recommended disease activity measures [28], and indirectly in the remainder (with patient global assessment scores and pain intensity scores strongly correlated [29]).

### Prescribing evidence

We searched Web of Science for observational studies (published from 2004 until July 2024) examining analgesic, gabapentinoid, DMARD, and corticosteroid prescribing using datasets with national/substantial regional coverage. We excluded studies of specific subpopulations only (e.g. pregnant women). As anti-depressants are usually prescribed for non-pain reasons, and literature on the use of cannabinoids in rheumatic diseases (including RA) has recently been systematically summarised [30], we did not consider these drug classes.

### Search terms

**Fig. 1:**
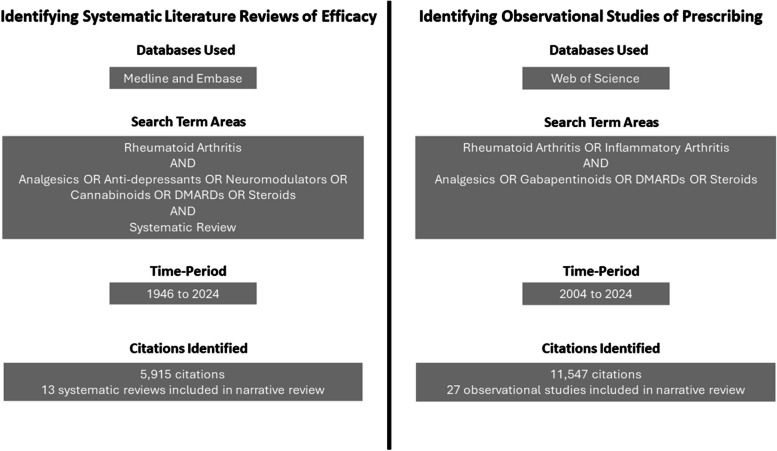
Overview of search strategy to identify systematic reviews of drug efficacy and observational studies of drug prescribing

Search terms are provided in supplementary data (Additional file 1: Tables S1 to S3) and an overview of the search strategy provided in Fig. 1. Database searching was conducted by one author (NC), with data extracted by two authors (ICS and NC). No language restrictions were applied.

## Systematic reviews of efficacy

### Reviews identified

**Fig. 2:**
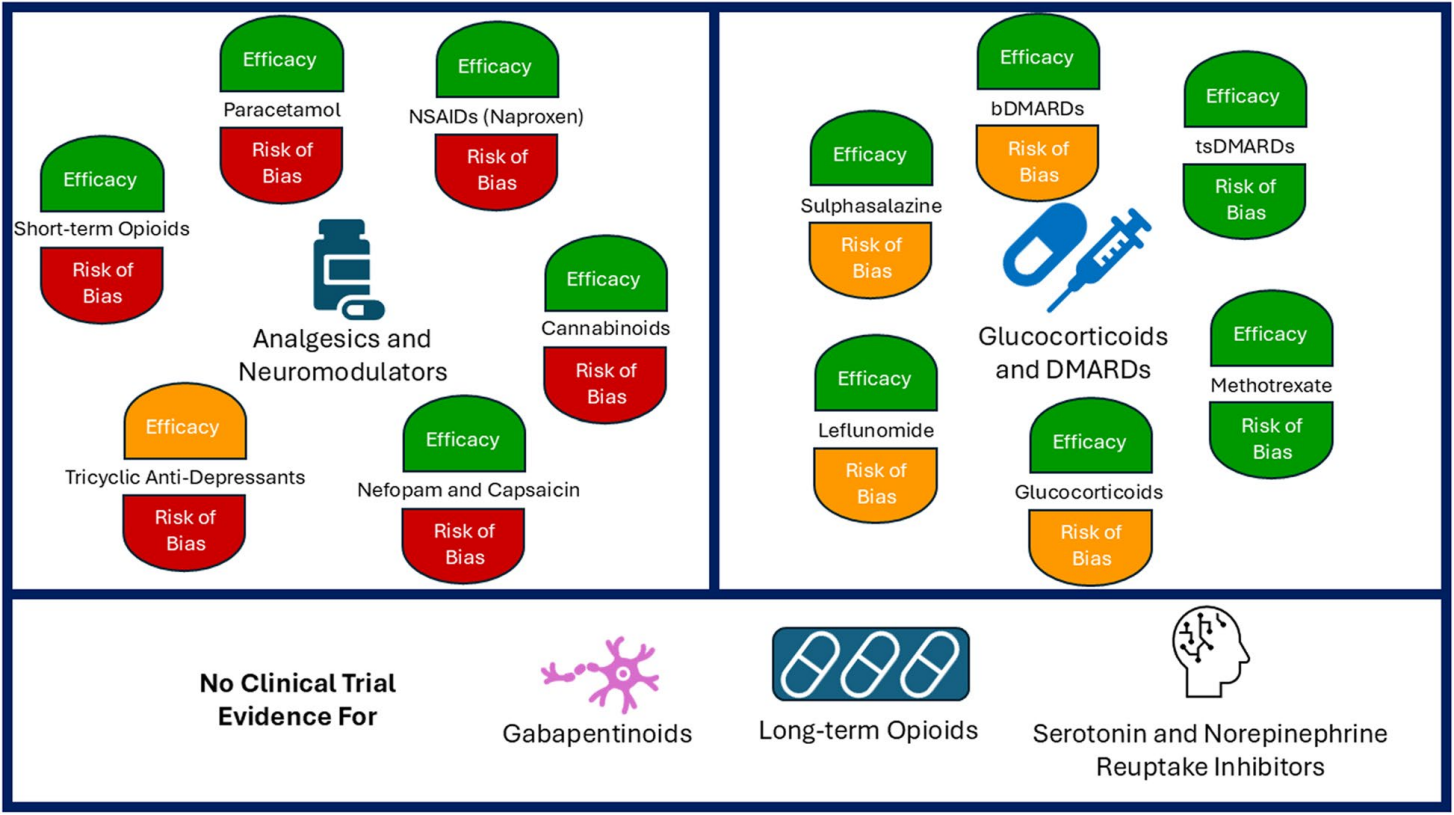
Summary of the evidence for efficacy and risk of bias from trials included in key systematic reviews of drugs for pain in patients with RA. Where efficacy is green, this indicates there is trial evidence indicating a favourable effect on pain (please see manuscript text and Table 1 for details on effect sizes). Where efficacy is orange, this indicates there is uncertain trial evidence for a favourable effect on pain. Where risk of bias is red, this indicates the trial evidence is at high risk of bias. Where risk of bias is orange, this indicates the trial evidence is at variable risk of bias. Where risk of bias is green, this indicates the trial evidence is at low risk of bias. Please see Table 1 for further details on each systematic review considered in this figure

From 5915 citations, we identified 34 relevant systematic reviews (with a further review identified from RA guidelines). Key reviews (totalling 13) for each drug class are described below, with a summary of their findings provided in Table 1 and Fig. 2. Details of all identified reviews are provided in supplementary data (Additional file 1: Table S4) [16, 31–63].

**Table 1:** Key systematic reviews reporting the efficacy of drugs for pain in patients with RA

Medicine class	Specific medicine	Systematic review	Year	Number of trials	Publication year range	Risk of bias	Duration of trials	Total number of patients	Key findings
*Analgesics*	Paracetamol	Hazlewood et al. [31]	2012	12 (9)	1959–1993	High	< 1 to 14 days	493 (412)	• Small benefits of paracetamol over placebo on “pain relief”
	Opioids	Whittle et al. [32]	2011	11 (3)	1969–2006	High	< 1 day to 6 weeks	672 (324)	• 18 more people (out of 100) reported good/very good improvement in symptoms with opioids (vs. placebo) • 30 more people (out of 100) reported adverse events with opioids (vs. placebo)
	NSAIDs	Paglia et al. [36]	2021	21 (13)	1986–2010	High	2 to 26 weeks	10,503 (6679)	• Naproxen 1000 mg provided significant reductions in pain vs placebo • No significant benefit observed for other NSAIDs
*Neuromodulators*	Gabapentinoids	NICE Guideline [16]	2008	0	-	-	-	-	• No trials identified
	Tricyclic anti-depressants	Richards et al. [39]	2011	8	1977–2000	High	< 1 day to 12 weeks	652	• Conflicting evidence of a benefit
	Nabiximols (cannabinoids)	Fitzcharles et al. [40]	2016	1	2006	High	5 weeks	58	• Significant improvement in pain on movement scores seen with active vs. placebo treatment
	Nefopam and Capsaicin	Richards et al. [38]	2012	3	1986–1991	High	2 to 4 weeks	52 Nefopam 31 Capsaicin	• Significant reduction in pain with nefopam vs. placebo after 2 weeks, but more adverse events • Greater reduction in pain with topical capsaicin vs placebo at 1 and 2 weeks, but 44% had application site burning
*Glucocorticoids*	Multiple	McWilliams et al. [62]	2021	33	1967–2017	Variable	1 week to 2 years	3123	• Significant benefit on pain intensity at 0 to 3 months
*Synthetic DMARDS*	Methotrexate	Lopez-Olivo et al. [41]	2014	7 (4)	1985–1999	Low	3 to 12 months	732 (468)	• Significantly lower pain scores with methotrexate at 3 to 12 months
	Sulphasalazine	Suarez-Almazor et al. [46]	2000	6 (3)	1983–1995	Variable	6 to 12 months	468 (179)	• Significantly lower pain scores with sulphasalazine at 6 to 12 months
	Leflunomide	Osiri et al. [45]	2003	33 (3)	1994–2007	Variable	6–24 months	6676 (724)	• Significantly lower pain scores with leflunomide at 6 months
*Biologic DMARDs*	Multiple	Jansen et al. [53]	2014	17 (13)	1999–2011^a^	Variable	24 weeks^b^	6477 (4211)	• Significantly greater reductions in pain with anti-TNF and tocilizumab
*Targeted synthetic DMARDs*	Multiple	Tóth et al. [61]	2022	50 (21)	2009–2020	Mainly Low^c^	< 6 months	24,135 (9588)	• Significantly lower pain scores with JAK inhibitors vs. placebo or biologic DMARDs

In the number of trials and number of patients’ cells, the number without parentheses refers to the number included in the overall review and the number in parentheses refers to the number included in the analysis of pain (or improvement in symptoms for opioids) as an outcome. a = two trials included in the meta-analysis were unpublished at the time of the systematic review; b = time-point for outcomes included in meta-analysis; c = three trials rated low, and one as “some concern” using the RoB 2 tool

### Paracetamol

Hazlewood et al. summarised paracetamol efficacy in 9 trials using a narrative synthesis (Table 1) [31]. All trials were short-term, used atypical dosing (doses ranging 650 mg to 7.5 g/day), and had high-risk of bias. Three (conducted in the 1970s; 6-h duration) compared single paracetamol doses to placebo using crossover designs, showing small, statistically significant improvements in “pain relief” (e.g. mean pain relief scores over trial period on a 0–3 scale of 1.2 with paracetamol vs. 0.8 with placebo); these also compared paracetamol with weak opioids, with no differences in efficacy found [64, 65]. Four trials compared paracetamol to NSAIDs (duration 1–2 weeks) [66–69], indicating a benefit of NSAIDs over paracetamol, although the relevance of this was uncertain as three either did not report effect sizes or significance levels. Two trials compared paracetamol with NSAIDs vs. NSAIDs [70, 71]; one showed no difference and the other significantly lower mean rest pain scores with paracetamol-naproxen combined vs. naproxen. The review authors concluded there was weak evidence for paracetamol’s efficacy.

### Opioids

A Cochrane review summarised opioid efficacy in 11 trials, all short duration (longest 6 weeks), in which the risk of bias was considered generally high [32]. Only two were published post-2000. Six studies with a duration of ≥ 1 week compared opioids to placebo; five reported superiority of opioids for at least one efficacy measure. Several studies could be included in meta-analyses for different outcomes. Three were included for the outcome of improvement in patient-reported global impression of clinical change of “good/very good”, with a pooled relative risk of 1.44 (95% CI 1.03, 2.03), equating to 18 more people out of 100 reporting a good/very good symptom improvement. Four were included for the outcome of adverse events, with the risk of experiencing at least one adverse event significantly more with opioids (pooled odds ratio 3.90; 95% CI 2.31, 6.56), equating to 30 more people out of 100 experiencing these. Evidence quality for these outcomes was “low”. The authors concluded there was limited evidence weak oral opioids may be effective for some patients, but adverse effects may offset benefits, with insufficient evidence to draw conclusions on opioids for > 6 weeks or strong opioids.

### NSAIDs

A network meta-analysis evaluated NSAID efficacy, including 21 trials of various NSAIDs of treatment durations ranging 2–26 weeks [36]. Thirteen were included in the meta-analysis for pain. Naproxen (1000 mg/day) associated with a statistically significant greater reduction in pain vs. placebo (standardised mean difference − 10.28; 95% CI − 20.39, − 0.17), although evidence was considered “very low” quality. No other NSAIDs had significant differences in their effects on pain vs. placebo/each other. Within this systematic review, only one trial of NSAIDs was considered to be at low risk of bias [72]. This represented a phase III, 12-week, randomised, double-blind, parallel-group trial comparing oral meloxicam of varying doses to placebo (negative control arm) and diclofenac 75 mg twice daily (active comparator arm). Eight hundred ninety four patients were randomised to treatment, with baseline endpoint scores similar amongst treatment groups. Statistically significant reductions in pain levels were seen for all active treatment groups compared to placebo (*P* < 0.05) with a mean reduction in 100 mm pain intensity VAS of − 21.2 (standard error [SE] 2.1), − 25.1 (2.1), and − 24.3 (2.1) with meloxicam 7.5 mg, 15 mg, and 22.5 mg daily, respectively; − 25.4 (SE 2.1) with diclofenac; and − 14.4 (SE 2.1) with placebo. As with many trials of NSAIDs in RA, however, patients were required to have been taking an NSAID pre-trial, and have flared on stopping it; consequently, the trial population is likely to include people most likely to benefit from using NSAIDs.

### Gabapentinoids

A National Institute for Health and Care Excellence (NICE) systematic review identified no trials examining gabapentin/pregabalin efficacy [16].

### Anti-depressants

A Cochrane review summarised anti-depressant efficacy in 8 trials (seven at high-risk of bias) [39]. All evaluated tricyclic anti-depressants. Due to poor-quality, heterogeneous trials with mixed results meta-analysis were deemed unsuitable. Qualitative analyses found no evidence of an effect of antidepressants on pain in the short term (< 1 week), and conflicting evidence of a medium- (1–6 weeks) or long-term (> 6 weeks) benefit.

### Cannabinoids

Fitzcharles et al. [40] examined the efficacy of cannabinoids in rheumatic diseases, identifying one trial (from 2006) of a cannabinoid administered as an oromucosal spray in RA [73]. It was considered at high risk of bias due to concerns regarding blinding of the outcome assessment, incomplete outcome data, and a small sample size (31 people randomised to active treatment). The primary outcome was pain on movement, measured using an 11-point numeric rating scale, with the baseline score (mean of last 4 days of 14-day baseline period) compared with endpoint score (mean of last 14 days of treatment). The median difference in change between baseline and endpoint scores was − 0.95 (95% CI − 1.83, − 0.02) with active vs. placebo treatment. More people receiving cannabinoids vs. placebo reported dizziness (26% vs. 4%) and light-headedness (10% vs 4%). A recent systematic review evaluated the association between cannabinoids and pain in people with “rheumatological conditions” more broadly, including both observational and trial data [30]. The authors conducted a meta-analysis of changes in pain scores before and after using cannabinoids, including six non-randomised studies (totalling 1079 patients). Four studies considered people with fibromyalgia, and two considered people with chronic pain related to a range of diagnoses. Whilst a statistically significant reduction in pain VAS scores was seen between baseline and follow-up assessments—pooled effect size of − 1.75 (95% CI − 2.75, − 0.76)—the non-randomised nature of included studies, alongside their consideration of non-RA populations, makes the relevance of their findings to RA pain management of uncertain significance.

### Nefopam and topical capsaicin

A Cochrane review identified two trials evaluating oral nefopam in RA (52 participants) and one evaluating topical capsaicin in RA and osteoarthritis (31 participants) [38]. All were considered at high-risk of bias. Meta-analysis identified a significant reduction in pain favouring nefopam over placebo (weighted mean difference − 21.2; 95% CI − 35.6 to − 6.7 after 2 weeks); however, nefopam associated with significantly more adverse events (relative risk 4.1; 95% CI 1.58 to 10.69). A significantly greater reduction in pain favouring topical capsaicin over placebo at 2 weeks was seen (mean difference − 34.4; 95% CI − 54.7 to − 14.1); whilst no separate safety data were available for patients with RA, 44% developed application site burning.

### Glucocorticoids

McWilliams et al. identified 33 trials examining systemic glucocorticoid efficacy at improving pain; 22 considered oral dosing [62]. Meta-analysis for the outcome of “spontaneous pain” at the earliest available timepoint showed a standardised mean difference of − 0.67 (95% CI − 0.84, − 0.50) for glucocorticoids vs. inactive comparators. Restricting analysis to 14 “high-quality” studies showed similar findings. Greatest improvement was seen in 100 mm pain visual analogue scale (VAS) at 0 to 3 months (mean difference − 15 mm; 95% CI − 20, − 9) followed by > 3 to 6 months (mean difference − 8 mm; 95% CI − 12, − 3), and > 6 months (mean difference − 7 mm; 95% CI − 13, 0). The authors concluded that systemic glucocorticoids are “analgesic in RA” with benefits greatest shortly after initiation.

### Synthetic DMARDs

Cochrane reviews have summarised the efficacy of methotrexate (seven trials) [41], sulphasalazine (six trials) [46], and leflunomide (33 trials) [45]. With methotrexate, differences in pain scores compared to placebo were reported in four studies (assessed at timepoints varying 12–52 weeks); the pooled mean difference for pain scores was − 2.02 (95% CI − 2.41, − 1.63) with methotrexate (267 participants) vs. placebo (201 participants) on a 0 to 10 scale. Trial risk of bias was generally low. With sulphasalazine, differences in pain scores compared to placebo were reported in three studies (assessed at timepoints varying between 6 and 12 months); the pooled mean difference for pain scores was − 8.71 (95% CI − 14.80, − 2.62) with sulphasalazine (84 participants) vs. placebo (95 participants) on a 0 to 100 scale. Study quality was assessed using the Jadad scale, ranging from 0 (worst) to 5 (best); two scored 5; one scored 4. With leflunomide, differences in pain scores compared to placebo were reported in three trials at 6 months; the pooled mean difference for pain scores was − 13.81 (95% CI − 15.91, 11.71) on a 0 to 100 scale with leflunomide (413 participants) vs. placebo (311 participants). Study quality was rated high in all three trials. Overall, these reviews provide generally high-quality evidence that synthetic DMARDs reduce pain intensity in active RA.

### Biologic DMARDs

A systematic review and network meta-analysis evaluated the efficacy of biologics for pain in RA [53]. This included 17 trials, all rated “good quality” (Jadad scores 3–5); 13 trials provided an outcome for pain. Relative to placebo, both anti-TNF (pooled estimate − 20.2; 95% CI − 17.4, − 0.37) and tocilizumab (pooled estimate − 31.3; 95% CI − 27.7, − 0.53) monotherapy demonstrated significantly greater reductions in pain over 24 weeks (on a 0 to 100 scale), which were considered larger than the minimal clinically important difference defined by the authors (10 units). Tocilizumab monotherapy associated with greater improvements in pain, compared to anti-TNF monotherapy (pooled estimate − 11.1; 95% CI − 21.3, − 0.1).

### Targeted synthetic DMARDs

Tóth et al. evaluated the efficacy of Janus Kinase (JAK) Inhibitors at improving patient-reported outcome measures, including pain, in RA [61]. Twenty-one trials were included for the outcome of pain at < 6 months (assessed using a 0–100 VAS) for JAK inhibitors vs. placebo: weighted mean difference was 15.3 mm lower (95% CI 13.2, 17.3) with active treatment (“moderate” certainty of evidence). Four trials were included for this outcome for JAK inhibitors vs. biologic DMARDs; all compared tofacitinib/baricitinib to adalimumab. Whilst these indicated a small but statistically significant improvement in pain with JAK inhibitors relative to adalimumab—weighted mean difference 4.4 mm lower (95% CI 2.2, 6.5) with JAK inhibitors (“moderate” certainty of evidence)—they also showed statistically significant improvements for a range of other outcomes (including remission rates, ACR20 responses, and CRP reductions) suggesting small global (as opposed to analgesic-specific) benefits with JAK inhibitors relative to adalimumab. A post hoc analysis of the RA-BEAM trial by Taylor et al. has, however raised the possibility that the JAK inhibitor, baricitinib, may exert its effects on pain via alternative pathways to adalimumab [74]. This trial randomised patients with active RA to baricitinib (487 patients), adalimumab (330 patients), and placebo (488 patients) plus methotrexate for 24 weeks (with patients receiving placebo, switched to baricitinib thereafter, with the overall trial lasting 52 weeks). Pain was evaluated using a 100-m VAS. This analysis compared pain level reductions at week-24 between treatment arms, stratified by CRP status, using analysis of covariance. It also used a mediation analysis to evaluate the extent to which these drugs reduce pain via reductions in inflammation levels (represented by ESR levels, CRP levels, and SJCs) and the extent to which they reduce pain via other effects. At week 24, statistically significant reductions in pain levels from baseline were seen for baricitinib in all CRP level categories (≤ 3, ≤ 10, and > 10 mg/L), but only for adalimumab in the CRP level category ≤ 3 mg/L. In the mediation analysis, changes in inflammation levels accounted for approximately 40% of pain improvements observed with baricitinib vs 50% of those observed with adalimumab. Whilst this analysis suggests the difference in analgesia between baricitinib and adalimumab cannot be solely accounted for by their differential effects on inflammation, as it represents a post-hoc analysis, it requires interpretation within this context.

### Summary

Systematic reviews show moderate- to high-quality trial evidence (from more contemporary time-periods) that, in patients with active RA, short-term systemic glucocorticoids and synthetic, biologic, and targeted synthetic DMARDs have efficacy at reducing pain relative to placebo. Conversely, they show low-quality evidence from trials in more historical time-periods that (aside from naproxen) analgesics and neuromodulators provide improvements in pain relative to placebo. No trials have evaluated gabapentinoids, serotonin and norepinephrine reuptake inhibitors, or long-term opioids, meaning that whilst no evidence exists to support their use for pain in RA, they cannot be entirely discounted as therapeutic options.

## Prescribing practice

### Studies identified

From 11,547 citations, our search identified 67 relevant observational studies. From these, we describe 21 key studies of analgesic prescribing and 6 of glucocorticoid and/or DMARD prescribing in this review (Table 2). For studies examining analgesic prescribing: 9 considered opioids, 5 NSAIDs, and 7 multiple analgesics. Geographically, they spanned North America, Australia, Columbia, Europe, Iceland, and Japan, with sizes ranging from 359 to 88,097 patients. For studies examining DMARD and glucocorticoid prescribing: 4 were conducted in North America, 1 in the UK, and 1 in Norway; study sizes ranged 829–71,411 patients. Details for the remaining observational studies are provided in supplementary data (Additional file 1: Tables S5 [20, 21, 75–106] and S6 [80, 83, 99, 102, 103, 107–134]).

**Table 2:** Key observational studies reporting analgesic prescribing levels or predictors in patients with RA

Authors	Year	Analgesics considered	Region	Size	Study aim	Key Findings
Baker et al. [84]	2021	Opioids	North America	37,868	Evaluate if incident chronic opioid use higher in patients with obesity	• 27% using opioids at baseline • Severe obesity associated with higher risk of chronic opioid use (HR 1.74; 95% CI 1.72, 2.04)
Black et al. [90]	2019	Opioids	Australia	3225	Determine factors associating with opioid use	• Population-averaged prevalence of opioid use 33%, and 62% reported ever-use over 5 years of follow-up • Opioid use higher in females and decreased with older baseline age
Curtis et al. [21]	2017	Opioids	North America	70,929	Examine trends over time and variability in individual physician prescribing of opioids	• In average rheumatologist’s practice, 40% of RA patients used prescription opioids regularly • Factors associated with regular use of opioids included younger age, female sex, African American race, back pain, fibromyalgia, anxiety, and depression • Patients cared for by the same physician 25% more likely to be regular opioid users
Huang et al. [81]	2021	Opioids	North America	4.5 million outpatient visits	Examine outpatient opioid prescribing practices and factors associated with opioid prescriptions in patient visits	• 24.3% of visits led to opioid prescriptions, which increased over time • Several factors (patient and provider) associated with opioid prescribing including age, ethnicity, and other medicines
Lee et al. [92]	2019	Opioids	North America	26,288	Examine trends in chronic opioid use in 2002 to 2015 and identify clinical predictors	• Chronic opioid use doubled from 2002 to 2015, from 7.4 to 16.9% • Pain and antidepressant use were strongest predictors of chronic opioid use
Lee et al. [88]	2020	Opioids	North America	9337	Examine whether a physician’s past opioid prescribing practice associates with long-term opioid use by their patients	• Clinician’s baseline opioid prescribing practice significant predictor of whether a patient will become a long-term opioid user
Machado-Duque et al. [86]	2020	Opioids	Columbia	1329	Determine trends in use of opioids	• 84.9% used opioids for at least 1 month • 46.7% used opioids for ≥ 12 months • Increasing age and other medicine use (e.g. antidepressants, biologics, glucocorticoids) associated with significantly increased risk of chronic opioid use
Navarro-Millan et al. [87]	2020	Opioids	North America	12,931 to 15,599	Determine racial/ethnic differences in use of opioids in people no longer working due to disability	• 61.1 to 63.8% were chronic opioid users (depending on the year considered) • Higher chronic opioid use in people of white ethnicity compared to other ethnicities
Park et al. [89]	2019	Opioids	North America	2330	Examine changes in opioid utilisation after initiation of anti-TNF	• Small decrease in overall opioid utilisation after anti-TNF initiation, falling from 54.0 to 51.0%
Baser et al. [100]	2013	NSAIDs	Turkey	2613	Estimate and identify determinants of direct medical costs associated with RA in Turkey. Costs examined over 12-month period following index date	• Most patients were prescribed NSAIDs over 12-month follow-up period (90% with incident and 93% with prevalent RA)
Crossfield et al. [83]	2021	NSAIDs	UK	71,411	Assess whether modern RA management has reduced long-term prescriptions of oral corticosteroids and NSAIDs	• Long-term NSAID prescribing declined from 45.9% of patients in 1998, to 25.1% in 2017
Hirata et al. [80]	2021	NSAIDs	Japan	6407	Investigate the prescribing trend patterns of biologic DMARDs	• 70.4% of patients receiving a biologic in 2012 were also prescribed an NSAID. This declined over time (last analysis year 2018)
Palsson et al. [75]	2024	NSAIDs	Iceland	359	Study the impact of anti-TNF therapy on the use of NSAIDs in patients with RA	• Mean annual daily doses of NSAIDs fell from 148 to 86 in the 2 years pre- and post-starting anti-TNF (by 42%)
Katada et al. [99]	2015	NSAIDs	Japan	5126	Investigate prescription patterns and trends for anti-rheumatic drug use	• In incident RA, more patients received first line treatment with NSAIDs than DMARDs
Accortt et al. [95]	2017	Opioids and NSAIDs	North America	6737	Evaluate healthcare utilisation before and after initiation etanercept	• Significant reduction in opioid (54.8 to 52.2%) and oral NSAID (50.8 to 39.4%) use pre- and-post therapy
Gadzhanova and Roughead [77]	2024	Opioids and NSAIDs	Australia	18,360	Examine analgesic use around the initiation of biologic DMARDs	• NSAID use was less in the 12 months before vs. 12 months after TNF-initiation (19 vs. 13%) although levels of use were declining significantly prior to anti-TNF initiation • Opioid use was similar (15 vs 17%)
Hunter et al. [82]	2021	Opioids, NSAIDs, neuromodulators, topical pain treatments, non-narcotic analgesics	North America	34,047	Compare analgesic use in incident RA vs controls over 2 years and examine changes in pain medication use pre/post-biologics	• 83.0% used pain medication(s) at baseline • NSAID and opioid use fell pre- vs post-biologic initiation (NSAIDs 61.1 vs. 41.5% and opioids 52.0 vs. 40.4%)
Jobski et al. [96]	2017	Opioids, NSAIDs, neuromodulators	Germany	3140	Assess pharmacological treatment with respect to pain and depression	• 65.5% received analgesics • Analgesic use increased with pain intensity levels • For all pain levels, analgesic use higher in those with moderate to severe vs. mild or no depressive symptoms
Kawai et al. [101]	2011	Opioids and NSAIDs	North America	32,476	Study association between DMARD initiation and co-therapy use	• DMARD initiation associated with proportion of NSAID users declining by 13%; the proportion of narcotic users changed little by 2.5%
Kern et al. [94]	2018	Opioids and “any pain medication”	North America	63,101	Examine treatment patterns in newly diagnosed patients	• 77.8% received any pain medication and 65.2% an opioid anytime post-index date
Scott et al. [20]	2024	Basic analgesics, opioids, NSAIDs, gabapentinoids	England	51,177 to 88,097	Describe analgesic prescribing and its variation and determine prognostic factors for chronic analgesic prescriptions	• From 2004 to 2020, NSAID use declined substantially, opioid use fell slightly, and gabapentinoid use increased • Prescribing of non-NSAID analgesics greatest in people living in areas of deprivation, older people, and females

### Analgesics

#### Prescribing levels

**Fig. 3:**
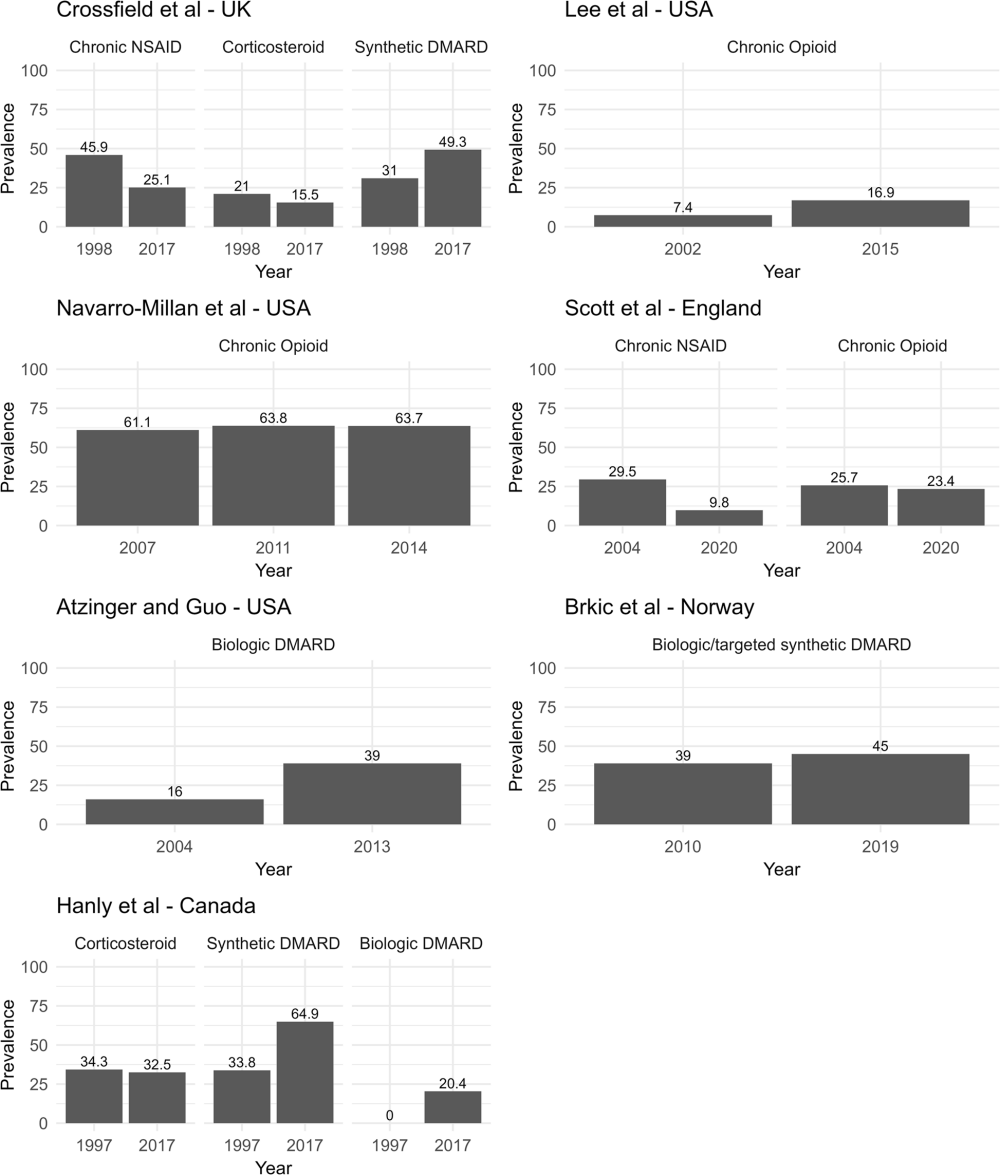
Observational studies reporting the prevalence of chronic analgesic, DMARD, and corticosteroid prescriptions or use over time in patients with RA. Different studies used different definitions of chronic prescriptions. Scott et al.: the denominator is 100 person-years, and the population includes patients with RA, psoriatic arthritis, and axial spondyloarthritis (although RA was the commonest diagnosis). Crossfield et al.: DMARD and corticosteroid prescriptions are long-term. Navarro-Millan et al.: the population comprises beneficiaries of Social Security Disability Insurance (no longer working because they are considered disabled) and < 65 years old. Studies included in the figure are those reporting the prevalence of chronic NSAID, chronic opioid, DMARD, and corticosteroid prescriptions/use in > one calendar year that contain extractable data

Substantial prescribing of all forms of analgesics across countries is seen (Table 2). For example, in a North American cohort study using health insurance data from 2006 to 2014 (involving 70,929 patients), regular opioid use (defined as ≥ 3 prescriptions or ≥ 90 days of cumulative use in each 12-month calendar interval) was observed in > 40% of patients every year [21]. In a Columbian cohort study, 84.9% of 1329 patients with RA used opioids for ≥ 1 month over 7 years [86]. In English electronic health record data (Clinical Practice Research Datalink Aurum) in each year from 2004 to 2020 approximately one in four received a long-term opioid prescription [20]. This study also demonstrated reductions in oral NSAID prescribing over 17 years, but rising gabapentinoid prescriptions (occurring in < 1% in 2004, and approximately 10% in 2020). Other studies in Japan and the UK also demonstrated declining NSAID prescriptions over time (although they remained common) with 25.1% receiving a long-term NSAID prescription in the UK in 2017 [83], and 80.9% of patients with incident RA in Japan receiving an NSAID as the main first line treatment in 2011 [99]. Figure 3 demonstrates the trends in chronic NSAID and opioid prescriptions/use (in studies containing extractable data from > one calendar year); substantial reductions in chronic NSAID prescriptions were observed in the UK and England, with chronic opioid prescriptions/use changing little.

#### Prescribing changes post-DMARD initiation

Several studies evaluated analgesic prescribing pre-/post-DMARD initiation, showing reductions, which were small for opioids. Park et al. reported that pre- and post-anti-TNF initiation, the proportion of 2330 patients receiving any opioid decreased from 54.0 to 51.0% [89], and Kawai et al. reported that amongst 32,476 patients initiating new DMARDs, in the 6–12 months afterwards the proportion receiving opioids decreased by 2.5% [101]. Larger reductions were reported for NSAIDs, with the study by Kawai et al. reporting a 12.9% reduction for NSAIDs, and another by Hunter et al. reporting that during the 12-month pre- and post-biologic DMARD initiation, NSAID prescriptions fell from 61.1 to 41.5% [82].

#### Prescribing variation

Analgesic prescribing varied substantially between groups of patients in England, with opioid and gabapentinoid prescriptions commoner in deprived areas and North England, older people, and females [20]. It also varied substantially between clinicians, with Curtis et al. reporting that patients cared for by the same physician were 25% (95% CI 18, 32) more likely to be regular opioid users or non-users, after controlling for other patient characteristics [21], and Lee et al. reporting that during follow-up, long-term opioid use occurred in 7.0% of patients of physicians with very low opioid prescribing rates, compared to 12.7% of patients of physicians with high prescribing rates [88].

#### Patient factors associating with prescriptions

Two longitudinal studies reported that more intense pain had significant associations with chronic opioid use. In North America, using survey data, amongst 26,288 individuals not taking opioids at baseline, cox proportional hazards models demonstrated that severe pain was a significant predictor of chronic opioid use (HR 2.53; 95% CI 2.19, 2.92) [92]. In a cohort study in the Australian biologics registry, within-patient opioid use associated with higher self-reported pain scores, which also associated with a higher probability of starting opioids and a lower probability of stopping them [90]. An increased body mass index (BMI) also has significant associations with opioid prescribing. In English primary care data, BMI at RA diagnosis had a significant association with the subsequent receipt of a chronic opioid prescription, with HRs (95% CI) of 1.66 (1.59, 1.73) and 1.24 (1.19, 1.29) for people categorised as overweight and obese, relative to those categorised as normal weight/underweight [20]. Baker et al. examined the association between BMI (at enrolment) and incident chronic opioid use in a longitudinal study using semi-annual surveys [84]. Severe obesity associated with a higher risk of overall (HR 1.74; 95% CI 1.72, 2.04) and strong (HR 2.11; 95% CI 1.64, 2.71) opioid use, compared to normal BMI. Other factors with significant associations with chronic opioid prescriptions include the presence of depression, anxiety, and fibromyalgia [20, 21].

### Glucocorticoids and DMARDs

Studies showed consistent findings, with the prescribing of DMARD types increasing over time, but long-term glucocorticoid prescribing remaining similar (Table 3). In UK primary care electronic health record data, Crossfield et al. reported that the percentage with incident RA receiving a long-term synthetic DMARD in the 12 months post-diagnosis rose from 41.6% in 1998, peaking at 67.9% in 2009 then falling slightly in 2016 to 54.7% [83]. The percentage receiving long-term corticosteroids changed little (22.2% in 1998; 19.1% in 2016). In Canada, Hanly et al. reported that (in health administrative data from 8240 patients) between 1997 and 2017 the percentage prescribed synthetic and biologic DMARDs in each year rose from 33.8 to 64.9% and 0 to 20.4%, respectively, but the percentage prescribed corticosteroids changed little (from 34.3 to 32.5%) [111]. Similarly, in the USA (within pharmacy claims data from 40,373 patients) the percentage prescribed biologic DMARDs rose from 16% in 2004 to 39% in 2013 [118], and in Norway (within data from 10 centres) the percentage receiving a biologic or targeted synthetic DMARD increased from 39% of 4909 patients in 2010 to 45% of 9335 patients in 2019 [110]. Crowson et al. compared glucocorticoid use between two cohorts of patients with RA: those diagnosed in 1999–2008 and those in 2009–2018 [109]. Glucocorticoids were initiated within 6 months of RA diagnosis in 67% of patients in 1999–2008 and 71% of patients in 2009–2018. Jeong et al. reported trends in biologic and targeted synthetic DMARD prescribing in the 664 days before and after the January 2021 release of trial data showing an increased risk of major cardiovascular events/cancer with JAK inhibitors; significant reductions and increases in the prescribing of JAK inhibitors and anti-TNF biologics, respectively, were seen [108]. Figure 3 highlights these time trends in glucocorticoid/DMARD prescribing.

**Table 3:** Key observational studies reporting long-term glucocorticoid and DMARD prescribing levels in patients with RA

Study	Years considered	Drugs	Region	Size	Key findings
Atzinger and Guo [118]	2004 to 2013	Biologic and targeted synthetic DMARDs	USA	40,473	• Prescriptions for biologic DMARDs increased more than 20% per patient from 2004 to 2013
Brkik et al. [110]	2010 to 2019	Biologic and targeted synthetic DMARDs	Norway	4909 to 9335	• Percentage receiving a biologic or targeted synthetic DMARD rose from 39% in 2010 to 45% in 2019
Crossfield et al. [83]	1998 to 2016	Glucocorticoids and synthetic DMARDs	UK	71,411	• Proportion receiving long-term synthetic DMARDs in the year post-diagnosis rose from 41.6% in 1998, peaking at 67.9% in 2009, then falling to 54.7% in 2016 • Proportion receiving long-term corticosteroids in the year post-diagnosis changed little, being 22.2% in 1998 and 19.1% in 2016
Crowson et al. [109]	1999 to 2018	Glucocorticoids	USA	829	• In patients diagnosed in 1999 to 2008, 67% initiated a glucocorticoid within 6 months, increasing to 71% diagnosed in 2009 to 2018 • Similar levels of glucocorticoid discontinuation rates within 6 months of initiation between these two groups (39.1 vs. 42.9%)
Hanly et al. [111]	1997 to 2017	Glucocorticoids, synthetic and biologic DMARDs	Canada	8240	• Proportion prescribed synthetic DMARDs and biologic DMARDs in each year rose from 33.8 to 64.9% and 0 to 20.4% • Proportion prescribed corticosteroids changed little from 34.3 to 32.5%
Jeong et al. [108]	2012 to 2022	Biologic and targeted synthetic DMARDs	USA	34,656	• Prescribing of JAK inhibitors reduced and of anti-TNF biologics increased in the 18 months post-January 2021 press release regarding the increased risks of major cardiovascular events and cancers with JAK inhibitors

### Summary

Many patients with RA receive analgesics (particularly long-term opioids), with prescribing levels varying substantially between clinicians and groups of patients. Whilst NSAID use has fallen over time, gabapentinoid prescribing has increased. DMARD prescribing levels have risen, but long-term glucocorticoid prescribing remains relatively static.

## Research agenda

Data summarised in this review show that drug prescribing for pain in RA fails to align with research evidence for efficacy—despite limited evidence for analgesics (and no evidence for long-term opioids/gabapentinoids) they are widely prescribed. Closing this evidence-to-practice gap requires the following research: (1) qualitative studies exploring prescribing drivers; (2) high-quality contemporary trials of analgesic efficacy in RA; (3) an increased focus on pain management in RA guidelines, with an associated implementation strategy.

### Qualitative research

In the context of opioids in chronic non-cancer pain, qualitative evidence syntheses using meta-ethnography show that key patient perspective themes are “reluctant users with little choice” (patients feeling there was no other choice available) and “understanding opioids” (patients reporting knowledge acquired gradually and ad hoc) [135], suggesting key drivers are a lack of available non-pharmacological treatment options and a lack of patient education on risks/benefits at opioid initiation. A key healthcare professional theme is “pain is pain” (people reporting a professional duty to resolve pain); this suggests a lack of prescriber knowledge on the relative inefficacy of opioids for chronic non-cancer pain is important [136]. Qualitative research in RA is urgently needed to explore the causes of widespread analgesic prescribing.

### Clinical trials

The lack of contemporary high-quality trial evidence that analgesics have/do not have efficacy at improving pain in RA seems an important barrier to changing practice. This is recognised in UK RA guidelines, which recommend research into analgesic effectiveness. The design of such trials is complicated by several factors. First, the widespread use of analgesics in RA means a placebo-controlled trial is unlikely to be feasible, and a withdrawal trial/active comparator design needed. Second, the close relationship between pain and disease activity [29] means that the latter factor needs accounting for either in design, analysis, or both; this is challenging, with sustained remission rare and disease activity varying between appointments. Third, as pain has substantial day-to-day variation in RA [137], the traditional use of end-point pain scores will not sufficiently capture patients’ pain experiences over time. An additional barrier is obtaining funding, as recognised in RA NICE guideline research recommendations, which comment that pharmacological funding for a trial of analgesics “is unlikely due to the drugs being generic and widely available”.

### Guidelines

Whist many RA guidelines exist, these focus on reducing disease activity using DMARDs, with the most recent American College of Rheumatology Guideline overlooking pain entirely [17]. Whilst these guidelines have been highly influential in facilitating treat-to-target care and high-cost biologic/targeted synthetic DMARD access, they fail to address the need of ensuring patients with RA receive evidence-based pain care. Within the UK, the British Society for Rheumatology is developing a pain management guideline for RA [138], which will be underpinned by an umbrella review of both pharmacological and non-pharmacological pain treatments (which was beyond the scope of this narrative review to undertake). There is a strong argument to address pain in either general RA guidelines or dedicated pain guidelines in other countries. To ensure these guidelines affect clinical practice, an associated implementation strategy is required, whose development is based on barriers/facilitators to changing practice at the level of patients, healthcare professionals, and organisations [139]. As detailed in a recent EULAR document on the implementation of recommendations in rheumatic diseases [139], effective implementation is complex, time-consuming, and difficult, with an umbrella review of complex intervention implementation strategies (most of which were clinical guidelines) reporting that whilst clinical champions, audit, and education were effective interventions, they had small effects [140].

## Conclusions

In patients with active RA, there is substantial evidence that DMARDs and short-term glucocorticoids improve pain. However, in a real-world setting many patients receiving DMARDs have persistent pain and receive long-term opioids and gabapentinoids despite absent trial evidence for efficacy. The reasons for this divergence between practice and evidence are not fully understood; they may reflect patients’ and clinicians’ beliefs about analgesics, which need evaluating in new qualitative research. As an absence of evidence does not mean analgesics are invariably ineffective, new high-quality trials of analgesic efficacy in contemporary RA populations are needed to better understand their relative benefits. Finally, there should be a greater focus on pain management within RA guidelines. In contrast to synovitis and inflammation—which can be simply and objectively measured in routine practice—the subjective, multi-factorial, and multidimensional nature of pain makes this a substantially more challenging outcome to improve.

## Supplementary Information

Additional file 1: Table S1. Search Terms used in Medline. Table S2. Search Terms used in EMBASE. Table S3. Search Terms used in Web of Science. Table S4. Systematic Reviews Examining Efficacy of Pharmacological Treatments for Pain in RA. Table S5. Observational Studies Examining Analgesic Prescribing/Use in Patients with RA. Table S6. Observational Studies Examining DMARD and Glucocorticoid Prescribing in Patients with RA.

## Data Availability

No datasets were generated or analysed during the current study.

## Abbreviations

BMI: Body mass index
CI: Confidence interval
DMARD: Disease-modifying anti-rheumatic drug
EULAR: European Alliance of Associations for Rheumatology
HR: Hazard ratio
VAS: Visual analogue scale
