# Supplementary material for: Pharmacological pain management in patients with rheumatoid arthritis: a narrative literature review
Source: BMC Med. 2025 Jan 29;23:54. doi: 10.1186/s12916-025-03870-0 (PMC11780779; doi:10.1186/s12916-025-03870-0)
Supplement: Supplementary file 1 — Additional file 1: Table S1. Search Terms used in Medline. Table S2. Search Terms used in EMBASE. Table S3. Search Terms used in Web of Science. Table S4. Systematic Reviews Examining Efficacy of Pharmacological Treatments for Pain in RA. Table S5. Observational Studies Examining Analgesic Prescribing/Use in Patients with RA. Table S6. Observational Studies Examining DMARD and Glucocorticoid Prescribing in Patients with RA. [file 12916_2025_3870_MOESM1_ESM.docx]

**Pharmacological Pain Management in Patients with Rheumatoid Arthritis: A Narrative Literature Review**

***Supplementary Data***

**Table S1. Search Terms used in Medline**

| **Search Term Number** | **Search Term** | **Number of Citations** |
| --- | --- | --- |
| 1 | exp Arthritis, Rheumatoid/ | 129483 |
| 2 | (rheumato* adj3 (arthrit* or diseas* or condition* or nodule*)).ti,ab,kf. | 137576 |
| 3 | (felty* adj2 syndrome).ti,ab,kf. | 786 |
| 4 | (caplan* adj2 syndrome).ti,ab,kf. | 144 |
| 5 | exp Analgesics/ | 604849 |
| 6 | exp narcotics/ | 149333 |
| 7 | exp Antirheumatic Agents/ | 479868 |
| 8 | opiate*.ti,ab,kf. | 26960 |
| 9 | opioid*.ti,ab,kf. | 124138 |
| 10 | non-steroidal anti-inflammator*.ti,ab,kf. | 20282 |
| 11 | NSAID*1.ti,ab,kf. | 31322 |
| 12 | ((cyclooxygenase* or COX*) adj3 (inhibit* or block*)).ti,ab,kf. | 29105 |
| 13 | (coxib* or cox-2i*).ti,ab,kf. | 1143 |
| 14 | Acetaminophen/ | 21369 |
| 15 | acetaminophen.ti,ab,kf. | 19412 |
| 16 | Paracetamol*.ti,ab,kf. | 14322 |
| 17 | exp Neurotransmitter Agents/ | 1441231 |
| 18 | neuromodulator*.ti,ab,kf. | 12900 |
| 19 | exp Anticonvulsants/ | 156832 |
| 20 | anticonvulsant*.ti,ab,kf. | 25671 |
| 21 | gabapentin*.ti,ab,kf. | 8543 |
| 22 | Gabapentin/ | 4547 |
| 23 | exp gamma-Aminobutyric Acid/ | 48946 |
| 24 | pregabalin.ti,ab,kf. | 4664 |
| 25 | exp Cannabinoids/ | 19327 |
| 26 | cannabinoid*.ti,ab,kf. | 26557 |
| 27 | cannabidiol.ti,ab,kf. | 6396 |
| 28 | cannabinol*.ti,ab,kf. | 845 |
| 29 | Tetrahydrocannabinol.ti,ab,kf. | 9677 |
| 30 | (THC or CBD).ti,ab,kf. | 19388 |
| 31 | delta-9-tetrahydrocannabinol.ti,ab,kf. | 4098 |
| 32 | delta-9-THC.ti,ab,kf. | 1184 |
| 33 | exp Antidepressive Agents/ | 163821 |
| 34 | antidepress*.ti,ab,kf. | 82136 |
| 35 | (anti adj1 depress*).ti,ab,kf. | 3646 |
| 36 | exp Selective Serotonin Reuptake Inhibitors/ | 47088 |
| 37 | (serotonin adj5 (inhibit* or block*)).ti,ab,kf. | 26877 |
| 38 | serotonin reuptake inhibitor*.ti,ab,kf. | 16141 |
| 39 | exp "Serotonin and Noradrenaline Reuptake Inhibitors"/ | 5679 |
| 40 | (norepinephrin* adj5 (inhibit* or block*)).ti,ab,kf. | 7699 |
| 41 | (noradrenalin* adj5 (inhibit* or block*)).ti,ab,kf. | 5446 |
| 42 | SSRI*.ti,ab,kf. | 12268 |
| 43 | SNRI*.ti,ab,kf. | 1908 |
| 44 | exp Monoamine Oxidase Inhibitors/ | 22944 |
| 45 | monoamine oxidase inhibitor*.ti,ab,kf. | 4777 |
| 46 | exp Immunosuppressive Agents/ | 351406 |
| 47 | immunosuppress*.ti,ab,kf. | 193336 |
| 48 | biosimilar*.ti,ab,kf. | 5781 |
| 49 | biologic*.ti,ab,kf. | 1160789 |
| 50 | (disease modifying adj5 (antir?eumatic* or anti r?eumatic*)).ti,ab,kf. | 8979 |
| 51 | DMARD*1.ti,ab,kf. | 7145 |
| 52 | csDMARD*1.ti,ab,kf. | 813 |
| 53 | cDMARD*1.ti,ab,kf. | 163 |
| 54 | bDMARD*1.ti,ab,kf. | 1556 |
| 55 | b?oDMARD*1.ti,ab,kf. | 11 |
| 56 | tsDMARD*1.ti,ab,kf. | 383 |
| 57 | tDMARD*1.ti,ab,kf. | 21 |
| 58 | sDMARD*1.ti,ab,kf. | 65 |
| 59 | ((tumo?r necrosis factor* or TNF*) adj3 (inhibit* or block*)).ti,ab,kf. | 28391 |
| 60 | (TNF?i or anti TNF*).ti,ab,kf. | 17101 |
| 61 | Janus Kinase Inhibitors/ | 1988 |
| 62 | ((janus kinase or JAK) adj3 (inhibit* or block*)).ti,ab,kf. | 8815 |
| 63 | JAKi*.ti,ab,kf. | 825 |
| 64 | (JAK1* or JAK2* or JAK3* or TYK2*).ti,ab,kf. | 19184 |
| 65 | (interleukin* or IL*).ti,ab,kf. | 1779740 |
| 66 | Interleukin 6/ | 79260 |
| 67 | glucocortico*.ti,ab,kf. | 86857 |
| 68 | corticosteroid*.ti,ab,kf. | 128882 |
| 69 | steroid*.ti,ab,kf. | 279921 |
| 70 | exp Glucocorticoids/ | 210899 |
| 71 | Or/1-4 | 176991 |
| 72 | or/5-70 | 5675711 |
| 73 | 71 AND 72 | 73887 |
| 74 | exp animals/ not humans/ | 5242260 |
| 75 | 73 not 74 | 69184 |
| 76 | meta-analysis/ | 205075 |
| 77 | meta analy*.ti,ab,kf. | 313167 |
| 78 | metaanaly*.ti,ab,kf. | 2866 |
| 79 | "systematic review"/ | 267881 |
| 80 | (systematic* adj3 review*).ti,ab,kf. | 368744 |
| 81 | systematic reviews.jn. | 2932 |
| 82 | (umbrella review* or (overview* adj3 systematic review*)).ti,ab,kf. | 3786 |
| 83 | Or/76-82 | 537014 |
| 84 | 75 and 83 | 2315 |

**Table S2. Search Terms used in EMBASE**

| **Search Term Number** | **Search Term** | **Number of Citations** |
| --- | --- | --- |
| 1 | exp rheumatoid arthritis/ | 249448 |
| 2 | (rheumato* adj3 (arthrit* or diseas* or condition* or nodule*)).ti,ab,kf. | 203768 |
| 3 | (felty* adj2 syndrome).ti,ab,kf. | 807 |
| 4 | (caplan* adj2 syndrome).ti,ab,kf. | 112 |
| 5 | exp analgesic agent/ | 13206628 |
| 6 | narcotic*1.ti,ab,kf. | 24491 |
| 7 | opiate*.ti,ab,kf. | 39186 |
| 8 | opioid*.ti,ab,kf. | 175150 |
| 9 | exp antiinflammatory agent/ | 265058 |
| 10 | non-steroidal anti-inflammator*.ti,ab,kf. | 29248 |
| 11 | NSAID*1.ti,ab,kf. | 57494 |
| 12 | ((cyclooxygenase* or COX*) adj3 (inhibit* or block*)).ti,ab,kf. | 37584 |
| 13 | (coxib* or cox-2i*).ti,ab,kf. | 1823 |
| 14 | paracetamol/ | 118811 |
| 15 | acetaminophen.ti,ab,kf. | 29276 |
| 16 | paracetamol.ti,ab,kf. | 24305 |
| 17 | exp "agents interacting with transmitter, hormone or drug receptors"/ | 3771046 |
| 18 | neuromodulator*.ti,ab,kf. | 16323 |
| 19 | exp anticonvulsive agent/ | 510881 |
| 20 | anticonvulsant*.ti,ab,kf. | 35785 |
| 21 | gabapentin*.ti,ab,kf. | 14548 |
| 22 | gabapentin/ | 41039 |
| 23 | pregabalin.ti,ab,kf. | 8271 |
| 24 | exp cannabinoid/ | 93474 |
| 25 | cannabinoid*.ti,ab,kf. | 35518 |
| 26 | cannabidiol.ti,ab,kf. | 8322 |
| 27 | cannabinol*.ti,ab,kf. | 1051 |
| 28 | Tetrahydrocannabinol.ti,ab,kf. | 12245 |
| 29 | (THC or CBD).ti,ab,kf. | 30802 |
| 30 | delta-9-tetrahydrocannabinol.ti,ab,kf. | 6144 |
| 31 | delta-9-THC.ti,ab,kf. | 2184 |
| 32 | exp antidepressant agent/ | 625151 |
| 33 | antidepress*.ti,ab,kf. | 118665 |
| 34 | (anti adj1 depress*).ti,ab,kf. | 6472 |
| 35 | (serotonin adj5 (inhibit* or block*)).ti,ab,kf. | 36556 |
| 36 | serotonin reuptake inhibitor*.ti,ab,kf. | 22620 |
| 37 | (norepinephrin* adj5 (inhibit* or block*)).ti,ab,kf. | 10086 |
| 38 | (noradrenalin* adj5 (inhibit* or block*)).ti,ab,kf. | 6559 |
| 39 | SSRI*.ti,ab,kf. | 21236 |
| 40 | SNRI*.ti,ab,kf. | 3598 |
| 41 | monoamine oxidase inhibitor*.ti,ab,kf. | 4510 |
| 42 | exp immunosuppressive agent/ | 1390344 |
| 43 | immunosuppress*.ti,ab,kf. | 305222 |
| 44 | biosimilar*.ti,ab,kf. | 11625 |
| 45 | biologic*.ti,ab,kf. | 1439595 |
| 46 | (disease modifying adj5 (antir?eumatic* or anti r?eumatic*)).ti,ab,kf. | 15551 |
| 47 | DMARD*1.ti,ab,kf. | 21112 |
| 48 | csDMARD*1.ti,ab,kf. | 2872 |
| 49 | cDMARD*1.ti,ab,kf. | 593 |
| 50 | bDMARD*1.ti,ab,kf. | 4927 |
| 51 | b?oDMARD*1.ti,ab,kf. | 47 |
| 52 | tsDMARD*1.ti,ab,kf. | 971 |
| 53 | tDMARD*1.ti,ab,kf. | 84 |
| 54 | sDMARD*1.ti,ab,kf. | 237 |
| 55 | ((tumo?r necrosis factor* or TNF*) adj3 (inhibit* or block*)).ti,ab,kf. | 43912 |
| 56 | (TNF?i or anti TNF*).ti,ab,kf. | 38956 |
| 57 | exp Janus kinase inhibitor/ | 36866 |
| 58 | ((janus kinase or JAK) adj3 (inhibit* or block*)).ti,ab,kf. | 15932 |
| 59 | JAKi*.ti,ab,kf. | 1585 |
| 60 | (JAK1* or JAK2* or JAK3* or TYK2*).ti,ab,kf. | 33642 |
| 61 | (interleukin* or IL*).ti,ab,kf. | 2355505 |
| 62 | interleukin 6/ | 450010 |
| 63 | glucocortico*.ti,ab,kf. | 120500 |
| 64 | corticosteroid*.ti,ab,kf. | 199866 |
| 65 | steroid*.ti,ab,kf. | 407303 |
| 66 | exp glucocorticoid/ | 900522 |
| 67 | Or/1-4 | 283808 |
| 68 | or/5-66 | 9715028 |
| 69 | 67 and 68 | 170210 |
| 70 | exp animal/ not human/ | 11804209 |
| 71 | 69 not 70 | 117263 |
| 72 | exp meta analysis/ | 325204 |
| 73 | meta analy*.ti,ab,kf. | 395964 |
| 74 | metaanaly*.ti,ab,kf. | 13870 |
| 75 | systematic review/ | 479665 |
| 76 | (systematic* adj3 review*).ti,ab,kf. | 448070 |
| 77 | systematic reviews.jn. | 2959 |
| 78 | (umbrella review* or (overview* adj3 systematic review*)).ti,ab,kf. | 4200 |
| 79 | or/72-78 | 782183 |
| 80 | 71 and 79 | 4945 |
| 81 | limit 80 to embase | 3600 |

**Table S3. Search Terms used in Web of Science**

| **Search Term Number** | **Search Term** |
| --- | --- |
| 1 | Rheumatoid arthritis |
| 2 | Inflammatory arthritis |
| 3 | RA |
| 4 | IA |
| 5 | Analgesic* |
| 6 | Non-Steroidal Anti-Inflammatory Drug* |
| 7 | NSAID* |
| 8 | Opioid* |
| 9 | Opiate* |
| 10 | Gabapentin* |
| 11 | Pregabalin* |
| 12 | DMARD* |
| 13 | Disease-Modifying Antirheumatic Drug* |
| 14 | Steroid* |
| 15 | Or/1-4 |
| 16 | Or/5-14 |
| 17 | 15 and 16 |

**Table S4. Systematic Reviews Examining Efficacy of Pharmacological Treatments for Pain in RA**

| **Drug(s)** | **Study** | **Publication Year** | **Key Findings** |
| --- | --- | --- | --- |
| Paracetamol | Hazlewood et al (31) | 2012 | - Please see manuscript table 1. |
| Opioids | Whittle et al (32) | 2011 | - Please see manuscript table 1. |
| NSAIDs | Atzeni et al (33) | 2021 | - Aim: systematic review and meta-analysis of RCTs to compare efficacy of ketoprofen and ibuprofen. - Four RCTs involving 456 patients met inclusion criteria. - Range of outcome measures used in meta-analysis: joint index; pain score; pain index; pain on pressure, - Meta-analysis showed statistically significant difference in efficacy in favour of ketoprofen (0.33, 95% CI 0.14-0.52, p = 0.0005) at all point-estimates of the mean-weighted size effect. |
| NSAIDs | Fidahic et al (34) | 2017 | - Aim: assess the benefits and harms of celecoxib in people with rheumatoid arthritis. - Included eight RCTs with durations of 4 to 24 weeks, published between 1998 and 2014 that involved a total of 3,988 adults. - Participants who received celecoxib reported less pain than placebo-treated people (11% absolute improvement; 95% CI 8% to 14%; NNTB = 4, 95% CI 3 to 6; 1 study, 706 participants). |
| NSAIDs | Garner et al (35) | 2017 | - Aim: assess the efficacy and toxicity of rofecoxib for treating RA. - Two RCTs evaluating rofecoxib for the treatment of RA were identified and met the inclusion criteria. One compared rofecoxib to placebo and was designed to assess the safety and efficacy of several doses of rofecoxib. The second trial compared rofecoxib to naproxen and was primarily designed to assess the safety of rofecoxib so did not include all the recommended RA efficacy measures. - Rofeboxib versus placebo: although results were presented only graphically, patients in the 25 and 50 mg groups had significantly greater mean change in pain scores. |
| NSAIDs and glucocorticoids | Paglia et al (36) | 2021 | - Please see manuscript table 1. |
| Combination analgesics | Ramiro et al (37) | 2012 | - Aim: assess efficacy and safety of combination pain therapy for people with inflammatory arthritis. - Twenty-three trials (total of 912 patients) met inclusion criteria (22 in RA and 1 in a mixed population of RA and osteoarthritis). - All except 1 were published before 1990. All trials were at high risk of bias, and heterogeneity precluded meta-analysis. - Statistically significant differences between treatment groups were reported in only 5/23 (22%) trials: in 3 trials combination therapy was better (2 trials with NSAID + analgesic versus NSAID only and 1 trial with 2 NSAIDs versus 1 NSAID), in 1 trial combination therapy was worse (opioid + neuromodulator versus opioid only), and in the fifth trial (NSAID + analgesic versus NSAID alone) reported results were mixed depending on the dosage used in the monotherapy arm. In general, there were no differences in safety and withdrawals due to inadequate analgesia between combination and monotherapy. - Conclusion: based on 23 trials, all at high risk of bias, there is insufficient evidence to establish the value of combination therapy over monotherapy for pain management in IA. Well-designed trials are needed to address this question. |
| NSAIDs, opioids, paracetamol, nefopam, gabapentinoids, anti-depressants. | NICE RA Guidelines (16) | 2014 | - In general, NSAID treatment seemed to provide some reduction in pain, but the results were often not sufficiently large to be considered clinically important. - Paracetamol plus opioid treatment was compared with placebo in 2 studies. The combined treatment failed to show a benefit over placebo for the outcome of pain. - Single small studies provided limited evidence for each of the following comparisons: tricyclic antidepressants versus placebo, NSAID versus paracetamol, and NSAID versus paracetamol plus opioid plus NSAID. The committee placed little weight on the highly limited, poor quality and inconsistent evidence for these comparisons. - No evidence was found for nefopam, gabapentinoids or SSRI and SSNRI antidepressants. |
| Neuromodulators | Richards et al (38) | 2012 | - Please see manuscript table 1. |
| Antidepressants | Richards et al (39) | 2011 | - Please see manuscript table 1. |
| Cannabinoids | Fitzcharles et al (40) | 2016 | - Please see manuscript table 1. |
| Synthetic DMARDs | Lopez-Olivo et al (41) | 2014 | - Please see manuscript table 1. |
| Synthetic DMARDs | Li et al (42) | 2013 | - Aim: assess efficacy of iguratimod for rheumatoid arthritis. - Four RCTs involving 1,407 patients were included. - The mean assessment of rest pain in the intervention groups vs. placebo was −0.71 lower (−0.89 to −0.54 lower) |
| Synthetic DMARDs | Steiman et al (43) | 2013 | - Aim: investigate the effect of non-biologic DMARDs on pain levels in IA - 33 eligible for inclusion: 9 in early RA, and 10 in established RA. - In early RA and established RA, all studies of DMARDs (monotherapy and combination therapies) consistently revealed statistically significant reductions. |
| Synthetic DMARDs | Katchamart et al (44). | 2010 | - Aim: evaluate efficacy of methotrexate monotherapy compared to methotrexate combination with non-biologic DMARDs in RA - 19 trials (2,025 patients) from 6,938 citations were grouped by the type of patients randomised. - Significant reductions of pain were found in the methotrexate combination group, but only in methotrexate -inadequate responders (absolute risk difference -9.72%, 95%CI -14.7% to -4.75%). |
| Synthetic DMARDs | Osiri et al (45) | 2003 | - Please see manuscript table 1. |
| Synthetic DMARDs | Suarez-Almazor et al (46) | 2000 | - Please see manuscript table 1. |
| Synthetic DMARDs | Suarez-Almazor et al (47) | 2000 | - Aim: estimate short-term effects of D-penicillamine for the treatment of rheumatoid arthritis - Six trials were identified, with 425 patients randomized to D-penicillamine and 258 to placebo. - A statistically significant benefit was observed for D-penicillamine when compared to placebo for all three-dose ranges and for most outcome measures including pain. - The standardized weighted mean differences between treatment and placebo in moderate doses was -0.56 (95% CI -0.87, -0.26) for pain. |
| Synthetic DMARDs | Suarez-Almazor et al (48) | 2000 | - Aim: estimate short-term efficacy of auranofin for the treatment of RA. - A statistically significant benefit was observed for auranofin when compared to placebo for pain. The weighed mean difference between treatment and placebo was -4.68 (95% CI -6.59, -2.77) for pain scores. |
| Synthetic DMARDs | Suarez-Almazor et al (49) | 2000 | - Aim: assess short-term effects of azathioprine for the treatment of rheumatoid arthritis. - Three trials with a total of 81 patients were included in the analysis - Forty patients were randomized to azathioprine and forty-one to placebo. - Pain outcomes were reported by only one trial, which reported a statistically significant improvement in pain assessments using change from baseline values: SMD ‐1.05 [95% CI ‐1.85, ‐0.25]. |
| Synthetic DMARDs | Shrestha et al (50). | 2020 | - Aim: compare efficacy of iguratimod with placebo and other DMARDs in adults with rheumatoid arthritis. - Identified 12 trials involving 1,938 participants. - Ten trials had an overall high risk of bias. - Pain intensity may be comparable between therapies. |
| Synthetic DMARDs and Biologic DMARDs | Ma et al (51) | 2019 | - Aim: conduct a network meta-analysis to compare and assess the efficacy and safety of 15 therapies employing biologics and DMARDs for RA. - A total of 67 randomized controlled trials with 20,898 patients met the inclusion criteria. - Tocilizumab and Tocilizumab + methotrexate showed better remission in pain compared to other treatments. |
| Synthetic DMARDs and Biologic DMARDs | Fleischmann et al (52) | 2017 | - Aim: estimate the efficacy of TNF inhibitor-methotrexate versus triple therapy in patients with RA. - Analysed 33 studies in patients with inadequate response to methotrexate and 19 in patients naive to methotrexate. - Patients with inadequate response to methotrexate: the two treatments were comparable for pain scores at 6 months |
| Biologic DMARDs | Jansen et al (53) | 2014 | - Please see manuscript table 1. |
| Biologic DMARDs | Zhou et al (54) | 2014 | - Aim: assess the efficacy of certolizumab in the treatment of RA. - Nine RCTs with a total of 5,228 patients were included in this meta-analysis, and all patients were administered certolizumab or placebo. - Four studies provided data for patient-reported outcome measures. Pooled results showed that certolizumab plus methotrexate significantly reduced the incidence of arthritis pain (RR = 4.00, 95% CI: 3.14, 5.09; P = 0.000) compared with controls. |
| Biologic DMARDs | Maxwell et al (55) | 2010 | - Aim: perform a systematic review of efficacy of abatacept in patients with rheumatoid arthritis. - Seven trials with 2,908 patients were included. - Patient-reported pain was significantly reduced in the abatacept group compared to placebo (mean pain in intervention groups on 100mm VAS = 10.71 lower (95% CI 12.97 to 8.45 lower)). |
| Biologic DMARDs | Mertens et al (56) | 2009 | - Aim: perform a systematic review of clinical effectiveness of anakinra in rheumatoid arthritis. - Five trials involving 2,846 patients, 781 randomized to placebo and 2,065 to anakinra, were included. - Visual analogue scale for pain scores demonstrated significant improvements with anakinra versus placebo at 24 weeks of treatment (data provided from one study): mean difference for change in pain of –0.10 (95% CI –0.15, –0.04). |
| Biologic DMARDs | Blumenauer et al (57) | 2002 | - Aim: assess efficacy of infliximab for the treatment of rheumatoid arthritis. - Two trials with a total of 529 patients met the inclusion criteria. - Mean difference for pain VAS for Infliximab/methotrexate vs. Placebo/ methotrexate (0-10cm) at 6 months (1 study only): ‐2.38 (95% CI ‐2.71, ‐2.05) |
| Biologic DMARDs and targeted synthetic DMARDs | Sparks et al (58) | 2023 | - Aim: assess real-world comparative effectiveness studies of biologic and targeted synthetic DMARDs in adults with rheumatoid arthritis - Most studies were multicenter observational cohort/registry studies (n = 60) and were published between 2011 and 2021 (n = 60). - The most common comparison was between TNF inhibitors and non-TNF inhibitor biologic DMARDs (35 studies). - Nine studies included data on patient pain. Of the studies that reported mean change from baseline in patient pain, there was a trend toward greater mean change with non-TNF inhibitor biologic DMARDs (tocilizumab and rituximab) compared with TNF inhibitors. - For studies that included a JAK inhibitor, there was no difference observed in mean change from baseline between baricitinib or tofacitinib and other biologic/targeted synthetic DMARDs. |
| Targeted synthetic DMARDs | Hernandez-Cruz et al (59) | 2023 | - Aim: describe the real-world clinical characteristics of baricitinib-treated patients with RA, prescription patterns, effectiveness, drug persistence, patient-reported outcomes, and safety of baricitinib. - Seventy studies were included, of which 40 were abstracts. - 14 studies observed the effect of baricitinib on pain - Overall, treatment with baricitinib was observed to rapidly improve pain in a way comparable to biologic DMARDs. |
| Targeted synthetic DMARDs | Song et al (60) | 2014 | - Aim: assess efficacy of tofacitinib in patients with active rheumatoid arthritis. - Five RCTs, including three phase-II and two phase-III trials involving 1,590 patients, met the inclusion criteria. - Weighted mean difference in pain VAS (3 studies) of –18.2 (95% CI –29.5, –8.23) with tofacitinib 10mg twice daily. |
| Targeted synthetic DMARDs | Toth et al (61) | 2022 | - Please see manuscript table 1. |
| Glucocorticoids | McWilliams et al (62) | 2021 | - Please see manuscript table 1. |
| Glucocorticoids and NSAIDs | Gotzsche et al (63). | 2004 | - Aim: determine whether short-term (i.e. as recorded within the first month of therapy), oral low-dose corticosteroids (corresponding to a maximum of 15 mg prednisolone daily) is superior to placebo and NSAID drugs in patients with rheumatoid arthritis - Ten studies, involving 320 patients, were included. Prednisolone had an effect over placebo on joint tenderness (standardised mean difference 1.30, 95% confidence interval 0.78 to 1.83), and pain (1.75, 0.87 to 2.64). - Prednisolone also had a greater effect than NSAID drugs on joint tenderness (0.63, 0.11 to 1.16) and pain (1.25, 0.26 to 2.24). |

**Table S5. Observational Studies Examining Analgesic Prescribing/Use in Patients with RA**

| **Drug** | **Study** | **Publication Year** | **Key Findings** |
| --- | --- | --- | --- |
| NSAIDs | Palsson et al (75) | 2024 | - Please see manuscript table 2. |
| Basic analgesics, NSAIDs, Opioids, Gabapentinoids | Scott et al (20) | 2024 | - Please see manuscript table 2. |
| Opioids | Huang et al (76) | 2024 | - Aim: to investigate opioid prescribing trends and assess the impact of the COVID-19 pandemic on opioid prescribing in rheumatic and musculoskeletal diseases (RMDs). - 1,313,519 RMD patients with opioid prescriptions and without cancer between 1 January 2006 and 31 August 2021 were examined. - New opioid users for RA increased from 2.6/10000 persons in 2006 to 4.5 in 2018 or 2019, followed by a fall to 2.4 in 2021. Prevalent opioid users for all RMDs increased from 2006 but plateaued or dropped beyond 2018. - During COVID-19 lockdowns, there were significant changes in the trend of prevalent opioid users for patients with RA, PsA and fibromyalgia. |
| NSAIDs, Opioids | Gadzhanova et al (77) | 2024 | - Please see manuscript table 2. |
| Opioids | Huang et al (106) | 2024 | - Aim: compare opioid prescribing among ambulatory visits with/without systemic autoimmune/inflammatory rheumatic diseases (SARDs) including RA, and assess factors associated with opioid prescribing. - Cross-sectional study using the National Ambulatory Medical Care Survey between 2006 and 2019. - SARDs group was more likely to be prescribed opioids (22.53%) than the non-SARDs group (9.83%) (adjusted odds ratio [aOR] 2.65; 95% CI 1.68-4.18). |
| Basic analgesics, NSAIDs, Opioids, Gabapentinoids | Scott et al (78) | 2022 | - Aim: to evaluate analgesic prescribing in English National Health Service-managed patients with IA. - Repeated cross-sectional analyses in the Consultations in Primary Care Archive (2000 to 2015) evaluated the annual prevalence of analgesic prescriptions. - In all forms of IA, in all years, most (65.3–78.5%) cases received analgesics, compared with (37.5–41.1%) controls. Opioid prescribing in cases fell but remained common (45.4% and 32.9% received at least 1 and ≥3 opioid prescriptions, respectively, in 2015). Gabapentinoid prescription prevalence in cases increased from 0% in 2000 to 9.5% in 2015, and oral NSAID prescription prevalence fell from 53.7% in 2000 to 25.0% in 2015. - Analgesic prescribing was commoner in RA than PsA/axial SpA, and 1.7–2.0 times higher in cases than controls. |
| NSAIDs | Lee et al (79) | 2022 | - Aim: comparison between early biologics treatment and late biologics treatment of RA patients in decreasing prescription days of glucocorticoids and analgesics using the Taiwan National Health Insurance Research database (1997 to 2013). - Use of steroids, DMARDs, and NSAIDs changed significantly after biologics treatment. Comparing before and after biologics treatment, oral medication was significantly tapered (all *P*<0.0001). |
| NSAIDs | Hirata et al (80) | 2021 | - Please see manuscript table 2. |
| Opioids | Huang et al (81) | 2021 | - Please see manuscript table 2. |
| Opioids, NSAIDs, neuromodulators, topical pain treatments, non-narcotic analgesics | Hunter et al (82) | 2021 | - Please see manuscript table 2. |
| NSAIDs | Crossfield et al (83) | 2021 | - Please see manuscript table 2. |
| Opioids | Baker et al (84) | 2021 | - Please see manuscript table 2. |
| NSAIDs, Opioids | Albrecht et al (85) | 2021 | - Aim: investigate the prescription frequency of analgesics in persons diagnosed with RA, axSpA, PsA and SLE in 2019 using claims data. - Metamizole (29–33%) was the most commonly prescribed analgesic. - In all patients 11–13% were prescribed weak and 6–8% strong opioids. From 2005 to 2019, the proportion of persons with an opioid prescription remained stable, with similar or slightly decreasing proportions of weak opioids and more frequent prescriptions of strong opioids. - The proportion of long-term opioid prescriptions in RA increased from 2006 to 2019 from 8.9% to 11.0%. |
| Opioids | Machado-Duque et al (86) | 2020 | - Please see manuscript table 2. |
| Opioids | Navarro-Millán et al (87) | 2020 | - Please see manuscript table 2. |
| Opioids | Lee et al (88) | 2020 | - Please see manuscript table 2. |
| Opioids | Park et al (89) | 2019 | - Please see manuscript table 2. |
| Opioids | Black et al (90) | 2019 | - Please see manuscript table 2. |
| Opioids | Bedene et al (91) | 2019 | - Aim: to determine prevalence of opioid prescriptions and adverse events associated with opioids and identify risk factors associated with opioid prescriptions in the Dutch population. - Cohort study using nationwide data to determine opioid prescription patterns and survey data. - 4.9% of the total population were prescribed an opioid in 2013, and 6.0% in 2017. Rheumatoid arthritis or fibromyalgia (OR, 3.77 [95% CI, 3.65-3.90]) were associated with opioid prescriptions. |
| Opioids | Lee et al (92) | 2019 | - Please see manuscript table 2. |
| Opioids | Chen et al (93) | 2019 | - Aim: to examine long-term prescription opioid use among patients with RA, SLE, PsA and AS, compared with patients with hypertension. - Cohorts with rheumatic disease were identified from a US commercial claims database (2003–2014) and matched with patients with hypertension. - Proportion of patients receiving long-term opioid prescriptions, and other measures of opioid prescriptions were higher among rheumatic disease cohorts. |
| Opioids and “any pain medication” | Kern et al (94) | 2018 | - Please see manuscript table 2. |
| NSAIDs, Opioids | Accortt et al (95) | 2017 | - Please see manuscript table 2. |
| Opioids | Curtis et al (21) | 2017 | - Please see manuscript table 2. |
| Opioids, NSAIDs, neuromodulators | Jobski et al (96) | 2017 | - Please see manuscript table 2. |
| Opioids | Zamora-Legoff et al (97) | 2016 | - Aim: to examine trends of opioid use in patients with RA. - Retrospective prescription data was examined from 2005 to 2014. 501 patients with RA and 532 non-RA subjects were included. - Total and chronic opioid use in 2014 was 40% RA vs 24% non-RA and 12% RA vs. 4% non-RA, respectively. Opioid use increased by 19% (95 % confidence interval [CI] 1.15, 1.25) per year in both cohorts. - RR of chronic opiate use for RA patients compared to non-RA subjects was highest in adults aged 50-64 years (RR 2.82; 95 % CI 1.43-6.23). |
| Opioids | Kuo et al (98) | 2016 | - Aim: to assess temporal and geographic trends in rates of opioid prescription and relationship to opioid toxicity and different state regulations in Part D Medicare recipients (2007-2012). - Prolonged opioid use was associated with rheumatoid arthritis alongside older age, female gender, white race, low-income, living in a lower education area, and comorbidity of drug abuse, depression. |
| NSAIDs | Katada et al (99) | 2015 | - Please see manuscript table 2. |
| NSAIDs | Baser et al (100) | 2013 | - Please see manuscript table 2. |
| NSAIDs, Opioids | Kawai et al (101) | 2011 | - Please see manuscript table 2. |
| NSAIDs, Opioids | Grijalva et al (102) | 2008 | - Aim: to examine changes in patterns of medication utilization in patients with RA. - Data from Tennessee Medicaid databases (1995-2004) were used to identify adults with both a diagnosis of RA and at least one DMARD prescription each year. - 23,342 patients with treated RA were analysed. Utilization of glucocorticoids decreased from 46% to 38% (P < 0.001), whereas NSAID utilization increased from 33% to 38% (P < 0.001), and use of narcotics increased from 38% to 55% (P < 0.001). |
| NSAIDs | Goycochea-Robles et al (103) | 2007 | - Aim: to describe prescription practices and the degree of disease control in a large sample of patients with RA and AS treated by rheumatologists in Mexico. - 1208 RA and AS patients completed a self-administered questionnaire across the country. - In RA patients, 88.1% were treated with NSAIDs. |
| Opioids | Solomon et al (104) | 2006 | - Aim: to examine patterns of chronic opioid use in selected groups with arthritis and low back pain and compare them with patterns among persons with ischemic heart disease. - Selected patients (with RA, OA, chronic low back pain, or ischemic heart disease since 1995) were identified from a study database of Medicare beneficiaries. - 4% of subjects with rheumatoid arthritis used opioids chronically in 2001 (<1% in the other groups). No increase in the chronic use of opioids over the 6-year study period. - Low-potency opioids were the most commonly prescribed preparations for chronic users from all patient groups. |
| NSAIDs | Helin-Salmivaara et al (105) | 2005 | - Aim: to study prescribing of NSAIDs with corticosteroids, oral anticoagulants or SSRIs, as well as the use of gastroprotection among continuous and non-continuous users of NSAIDs in Finland. - A nested case-control study in a population-based cohort of NSAID users in 2000 was conducted using data in the National Prescription Database. - Of patients using continuous NSAIDs with oral corticosteroids, 73.3% had RA. |

**Table S6. Observational Studies Examining DMARD and Glucocorticoid Prescribing in Patients with RA**

| **Drug** | **Study** | **Publication Year** | **Key Findings** |
| --- | --- | --- | --- |
| Synthetic DMARDs | Huang et al (107) | 2023 | - Aim: examine factors associated with adding another DMARD in RA patients initiating MTX. - Retrospective cohort study using MarketScan data (2012-2014) involving adults (aged ≥18) with RA initiating an MTX (index date) between Jul 1, 2012 and Dec 30, 2013. - Among 8,350 RA patients starting MTX, 31.9% initiated any DMARD within the 1-year post-index period. - Among RA patients initiating a DMARD prescription after starting MTX, 945 (11.3%) received combination therapy with treatment addition of a DMARD to MTX; the majority added TNFi (58%), followed by a synthetic DMARD (37%); non-TNF biologic (4%), or tsDMARD (0.3%). |
| Biologic and targeted synthetic DMARDs | Jeong et al (108) | 2023 | - Please see manuscript table 3. |
| Glucocorticoids | Crowson et al (109) | 2023 | - Please see manuscript table 3. |
| Biologic and targeted synthetic DMARDs. | Brkic et al (110) | 2022 | - Please see manuscript table 3. |
| Glucocorticoids, synthetic and biologic DMARDs. | Hanly et al (111) | 2021 | - Please see manuscript table 3. |
| All DMARD types. | Perrone et al (112) | 2021 | - Aim: evaluate treatment patterns and pharmaco-utilisation of patients with RA in real-world settings in Italy. - Retrospective observational analysis was based on administrative databases of selected Italian entities. - All adult patients with RA diagnosis confirmed by ≥1 discharge diagnosis of RA or an active exemption code were enrolled in 2019. - 47,711 RA patients were identified. - As a first-line prescription in 2019, 43.2% were prescribed synthetic DMARDs, 5.2% bDMARDs and 0.3% baricitinib. |
| Glucocorticoids | George et al (113) | 2021 | - Aim: evaluate variability in glucocorticoid prescribing across rheumatologists to inform interventions to limit long-term glucocorticoid use to the lowest dose necessary. - Two cohorts created using Medicare data from 2006 to 2015. - In cohort 1 (RA patients receiving DMARDs), calculated each rheumatologist's "provider preference" for glucocorticoids (frequency of use compared to other providers), using the ratio of observed to expected number of patients receiving glucocorticoids to account for case mix. - In cohort 2 (RA patients receiving stable DMARD therapy), evaluated whether provider preference for glucocorticoids could independently predict use of ≥5 mg/day of glucocorticoids 6-9 months after initiation of DMARD therapy. Provider preference was highly variable, with physicians at the lowest and upper quartiles prescribing glucocorticoids 33% less often to 31% more often. - In cohort 2, provider preference was strongly associated with glucocorticoid use ≥5 mg/day at 6-9 months. |
| Biologic DMARDs | Sánchez‑Piedra et al (114) | 2021 | - Aim: assess changes in demographic characteristics, disease activity and treatment patterns in patients with rheumatoid arthritis who started a first- or second-line biologic between 2007 and mid-2020. - Patients diagnosed with RA included in the BIOBADASER (Spanish biologic) registry from January 2007 to July 2020 were included. - The use of TNF inhibitors as a first-line biologic treatment decreased, with a temporal trend towards the use of biologic DMARD monotherapy. |
| Glucocorticoids and synthetic DMARDs | Crossfield et al (83) | 2021 | - Please see manuscript table 3. |
| Biologic DMARDs | Hirata et al (80) | 2021 | - Aim: investigate prescribing trends of bDMARDs for patients with RA in Japan. - Descriptive study from 2012 to 2018 using the JMDC Claims Database, a nationwide claims database - Data of 6407 patients with rheumatoid arthritis extracted. - Proportion of patients prescribed biologic DMARDs was 1.0 per 1000 people and increased significantly over time (p < 0.0001). Additionally, the concomitant proportions of methotrexate (p < 0.0001), non-steroidal anti-inflammatory drugs (p < 0.0001) and glucocorticoids (p = 0.0001) prescribed with bDMARDs decreased significantly over time. |
| Targeted synthetic DMARDs. | Park et al (115) | 2021 | - Aim: compare trends in use of targeted disease-modifying anti-rheumatic drugs for rheumatoid arthritis between Korea and Australia. - Using sampled claims databases in Korea and Australia (2010 - 2018), analyzed trends in the use of individual targeted DMARDs (biologic and targeted synthetic) for RA in both countries. - The use of targeted DMARDs for the management of RA showed an increase of over 200 and 300% in Australia and Korea, respectively. |
| Synthetic and biologic DMARDs | Steffen et al (116) | 2018 | - Aim: provide picture of patterns of initiation of DMARDs in patients with newly diagnosed RA. - Ambulatory drug prescription data and physician billing claims data covering 87% of the German population, assembled a cohort of incident RA patients aged 15-79 years (n = 54,896) and assessed prescription frequency of total DMARDs within the first year of disease. 44% received a DMARD prescription within the first year of disease. - In multiple regression, younger patients (< 35 years) had 1.7-fold higher chances of receiving a synthetic DMARD than patients aged ≥ 65 years and almost tenfold higher chances to receive a biologic DMARD. |
| Synthetic and biologic DMARDs | Fakhouri et al (117) | 2018 | - Aim: describe treatment patterns and drug utilization profile among adult patients with RA. - Retrospective cohort analysis, using administrative databases of six Local Health Units in Italy, was performed. - All adult patients with a confirmed diagnosis of RA between January 1, 2010 and December 31, 2014 were enrolled. - Date of first RA diagnosis represented the index date for each patient. - 10,401 patients with RA were included. - 67% of patients were untreated at index date. - During follow-up 67.8% treated with biologic agents were persistent with initial therapy, compared to 45.7% for patients on synthetic DMARDs. |
| Biologic and targeted synthetic DMARDs. | Atzinger and Guo (118) | 2017 | - Please see manuscript table 3. |
| Synthetic and biologic DMARDs | Donges et al (119) | 2017 | - Aim: characterise use and costs of subsidising conventional DMARDs and biologic DMARDs in Australia from 2004-2014 through pharmaceutical benefits schemes. - Dispensing and expenditure data on conventional and biologic DMARDs were extracted from Medicare Australia and temporal trends analysed. - Conventional and biologic DMARD use increased 74% over the study period (4.86 to 8.46 defined daily dose [DDD]/1,000 population/day; average annual increase 6.7%). Conventional DMARDs accounted for the vast majority of total use and increased 55% (4.81 to 7.43 DDD/1,000 population/day), while biologic DMARD use increased 1,784% (0.055 to 1.030 DDD/1,000 population/day). |
| Biologic DMARDs | Desai et al (120) | 2017 | - Aim: describe time trends in use of biologic DMARDs in RA patients with private or public insurance in the United States. - Claims data from private (Optum Clinformatics, 2004-2015) and public (Medicaid Analytic eXtract [MAX], 2000-2010) insurance programs were used. - Patients with RA diagnosis codes and continuous health plan enrollment for 1-year baseline and 1-year follow-up periods were identified into 2 separate cohorts: (1) patients not using any biologic DMARD or (2) patients using a single biologic DMARD during the baseline period. - Initiation of the first biologic DMARD from group 1 and switch to a second bDMARD from group 2 was identified as the outcome of interest during the 1-year follow-up period.  113,031 RA patients with public insurance and 97,751 RA patients with private insurance were included in the study. - Rates of initiation of biologic DMARDs (per 100 patients) increased significantly over time in Medicaid data for incident RA patients (from 1.1 to 3.1, P = 0.0006) and prevalent RA patients (from 4.6 to 10.9, P = 0.008). - In Optum Clinformatics data, rates were stable, with 7.7 to 8.3 per 100 incident RA patients (P = 0.10) and 11.0 to 11.5 per 100 prevalent RA patients (P = 0.12). |
| Biologic DMARDs | Kalkan et al (121) | 2015 | - Aim: to test the hypothesis that physician preferences are an important determinant for prescribing biologic DMARDs for RA patients in Sweden. - Using data from the Swedish Rheumatology Quality Register, identified 4,010 RA patients who were not prescribed biologic DMARDs during the period 2008-2012, but who, on at least 1 occasion, had an synthetic DMARD and changed treatment for the first time to either a new synthetic DMARD or a biologic DMARD. - When adjusting for patient characteristics, disease activity, and the physician's local context, physician preference was an important predictor for prescription of biologic DMARDs. |
| Synthetic and biologic DMARDs | Katada et al (99) | 2015 | - Aim: to investigate prescription patterns and trends for anti-rheumatic drug use in Japanese patients with rheumatoid arthritis. - Used a large-scale claims database consisting of the medical claims of employee health insurance recipients, which included approximately one million insured people. - Claims data for incident 5,126 patients with diagnosis codes of RA between January 1, 2005 and October 31, 2011 were analyzed. - Number who received DMARDs including biologics as initial therapy was 629 (12.3 %), while the others received non-DMARD therapy only. - During the study period, use of methotrexate (MTX) and biologics as first-line drugs increased from 1.9 to 8.0 % and from 0 to 1.6 %, respectively (p < 0.001 for both), while that of non-steroidal anti-inflammatory drugs decreased (p = 0.004). |
| Glucocorticoids. | Yazdany et al (122) | 2014 | - Aim: investigate prevalence and predictors of receiving glucocorticoids alone for the treatment of RA in a nationwide sample of Medicare beneficiaries. - Among individuals ages ≥65 years with RA enrolled in the Part D prescription drug benefit in 2009, we compared those with ≥1 DMARD claim to those receiving glucocorticoid monotherapy, defined as no DMARD claim and an annual glucocorticoid supply of ≥180 days or an annual dose of ≥900 mg of prednisone or equivalent. - Of 8,125 beneficiaries treated for RA, 10.2% (n = 825) received glucocorticoids alone. - Beneficiaries with low incomes were more likely to receive glucocorticoids alone, as were those living in certain US regions. More physician office visits and hospitalizations were associated with glucocorticoid monotherapy. |
| Glucocorticoids. | Makol et al (123) | 2014 | - Aim: examine trends in glucocorticoid use and dosing among patients diagnosed with rheumatoid arthritis over time. - Population-based inception cohort of RA patients diagnosed during 1980-2007 followed longitudinally through medical records until death, migration, or December 31, 2008. - Study population comprised 349 patients diagnosed in 1980-1994 and 464 diagnosed in 1995-2007, with a median follow-up of 15.3 and 5.7 years, respectively. - A higher proportion of patients started glucocorticoids in their first year of disease in 1995-2007 (68% versus 36%; P < 0.001), but the starting dose (mean 8.7 versus 10.3 mg; P = 0.08) and cumulative dose in the first year of use (mean 1.8g [mean daily dose 4.9 mg] versus 2.1 gm [mean daily dose 5.8 mg]; P = 0.48) were not different. - A higher proportion also discontinued glucocorticoids in their first year of disease in the 1995-2007 cohort (P < 0.001). |
| Synthetic DMARDs. | Edwards et al (124) | 2012 | - Aim: describe current DMARDs prescriptions in RA and to identify temporal and regional trends in the UK. - Descriptive, register-based cohort study. - Permanently registered patients aged ≥18 years with a recorded diagnosis of RA between 1 January 1995 and 31 March 2010 and matched controls. Participants with RA were identified through screening of all patients in the General Practice Research Database (GPRD) with a clinical or referral record for RA and at least 1 day of follow-up. - From 35 911 patients in the full RA cohort, 15 259 patients (42%) had incident RA. - Analysis of prescribing in incident RA patients demonstrated that between 1995 (baseline) and 2010 there was a substantial increase in DMARD, and specifically methotrexate, prescribing across all regions with a less marked increase in combination DMARD prescribing. |
| Biologic and synthetic DMARDs | Harrold et al (125) | 2012 | - Aim: examine prescribing practices in the use of biologic and non-biologic DMARDs to treat patients with RA, before and after publication of the American College of Rheumatology (ACR) treatment recommendations. - Biologics-naive RA patients under the care of a rheumatologist in the US were identified from the Consortium of Rheumatology Researchers of North America registry. - After 1 visit, 24-37% of patients receiving methotrexate monotherapy who had moderate disease activity and a poor prognosis or high disease activity received care consistent with the ACR recommendations; after 2 visits, 34-56% of the MTX monotherapy group received care consistent with the recommendations. - In the patients receiving multiple nonbiologic DMARDs, 31-47% of those with moderate or high disease activity received care consistent with the recommendations after 1 visit, and 43-51% received such care after 2 visits. Publication of the recommendations did not significantly change treatment patterns for those with active disease. |
| Synthetic DMARDs | Rantalaiho et al (126) | 2011 | - Aim: determine which DMARDs are currently used by Finnish rheumatologists to treat early RA. - Information on sex, date of birth, and date of special medicine reimbursement decision for all new RA patients was collected from a nationwide register maintained by the Social Insurance Institution from 1 January 2000 to 31 December 2007. - Patient cohorts were registered in 2-year time periods (2000-01, 2002-03, 2004-05, 2006-07) and DMARDs purchased by the patient cohorts during the first year after the date of reimbursement decision for RA were registered. - 14,878 patients were identified. - Between 2000 and 2001 the most commonly used treatment strategy for early RA during the first 3 months was single DMARD treatment (56.1%) and the most commonly used DMARD during the first year was sulfasalazine (63.0%) - Between 2006 and 2007 the respective treatments were combination DMARDs (55.3%) and methotrexate (69.0%). - The change in treatment strategies as well as in DMARDs used was highly significant (p < 0.001 for linearity). - At the end of the study period only 4.9% of the patients with early RA were not receiving DMARDs during the first 3 months. |
| Synthetic DMARDs and glucocorticoids | Nikolaisen et al (127) | 2009 | - Aim: evaluate current use of DMARDs in the management of patients with early RA in Norway. - Observational multicentre study registering the type of therapy initiated in 820 DMARD-naive patients with early active RA. - Methotrexate monotherapy was selected for 78% of patients. Concurrent steroid therapy was started in 73% of patients, regardless of the type of DMARD therapy initiated. |
| Biologic DMARDs | Söderlin and Geborek (128) | 2008 | - Aim: study prescription patterns of biological treatment in rheumatoid arthritis patients in southern Sweden. - Rheumatologists in southern Sweden contribute to a voluntary register on the use of biologics in treating arthritis patients. This register covers >90% of all the prescriptions of biologics for arthritis patients in the region. The treatment of 1,839 patients was recorded in the register during 1999–2006. - RA patients were treated with biologics earlier, but only 16% of the patients received biologics within 2 years of disease onset in 2006. The percentage of RA patients who were prescribed biologics after only one previous non-biological DMARD (disease-modifying anti-inflammatory rheumatic drug) was 27% in 2006. Thirty-five per cent of all RA patients changed from one biological treatment to another. |
| Synthetic DMARDs, biologic DMARDs, and glucocorticoids. | Grijalva et al (102) | 2008 | - Aim: examine changes in patterns of medication utilization in patients with RA. - Data from Tennessee Medicaid databases (1995–2004) were used to identify adults with both a diagnosis of RA and at least one DMARD prescription each year. - Records from 23,342 patients with treated RA were analysed. - The proportion of patients who had a current DMARD prescription on the index date increased from 62% in 1995 to 71% in 2004 (P < 0.001). - Methotrexate was the most commonly used DMARD. - By the end of 2004, 22% of patients had a current prescription for a biologic, and etanercept represented 51% of all biologic therapies. During the study period, the overall utilization of glucocorticoids decreased from 46% to 38% (P < 0.001). |
| Synthetic DMARDs, biologic DMARDs, and glucocorticoids. | Khanna and Smith (129) | 2007 | - Aim: assess utilisation and costs of RA-related medical services and prescription medications among recipients enrolled in a state Medicaid program. - Retrospective, cross-sectional, descriptive analysis of West Virginia (WV) Medicaid fee-for-service administrative claims data. - 1157 recipients had >=1 medical service claim (hospitalization, emergency department visit, or office visit) with a primary diagnosis of RA. - 67.8% had >=1 prescription claim for an oral steroid, 40.1% for a synthetic DMARD, and 12.4% for a biologic agent. |
| Synthetic DMARDs, biologic DMARDs, and glucocorticoids. | Goycochea-Robles et al (103) | 2007 | - Aim: describe prescription practices in a large sample of patients with RA treated by rheumatologists in Mexico. - Board-certified Mexican rheumatologists across the country were asked to assess consecutive RA patients. - Of the 1096 RA patients, 93.3% were treated with synthetic DMARDs, 33.4% with steroids, and 6.4% with biological agents. |
| Synthetic DMARDs | Carli et al (130) | 2006 | - Aim: characterise temporal trends and factors associated with the prescription of DMARDs at the initial consultation in early RA. - Data from 2,584 patients with early RA at 19 hospitals were extracted from the Swedish Rheumatoid Arthritis Register for the period 1997-2001. - DMARD prescriptions, particularly for methotrexate, increased from 1997 to 2001 independently of patient characteristics. - Stratification by hospital type showed that patients in district hospitals were less likely to be prescribed DMARDs than those in university hospitals, independently of confounding factors. |
| Synthetic DMARDs and biologic DMARDs. | Gibofsky et al (131) | 2006 | - Aim: provide snapshot of use patterns, effectiveness, and safety of DMARDs, biologics, and combination therapies used to manage RA in clinical practice. - Patients with RA requiring a new DMARD or biologic (addition or switch) were eligible for the RADIUS study. Two separate patient cohorts were enrolled; RADIUS 1 patients initiated any new therapy at entry, and RADIUS 2 patients initiated etanercept at entry. - RADIUS 1 enrolled 4,959 patients, and RADIUS 2 enrolled 5,102 patients, mostly at community private practices. - In RADIUS 1, most patients initiated methotrexate monotherapy, followed by methotrexate in combination with a biologic or other DMARD. - In RADIUS 2, most patients initiated etanercept in combination with methotrexate followed by etanercept monotherapy. - When a new therapy was required, physicians tended to add another therapy versus switching therapies. |
| Glucocorticoids. | Thiele et al (132) | 2005 | - Aim: describe current use of glucocorticoids in German patients with RA. - Analysed data from 10,068 outpatients with RA from the national database of the German Collaborative Arthritis Centres for the year 2001 collected by more than 80 rheumatologists in hospitals and private practices. - Systemic glucocorticoid therapy was prescribed for 60% of all patients with RA in rheumatologic care. - The proportion of patients receiving systemic glucocorticoids in addition to DMARD therapy ranged from 53% to 81% of patients for the various DMARDs. - Glucocorticoid therapy was administered more often in combination with tumour necrosis factor inhibitors (81%), cyclosporin A (80%), or leflunomide (77%) than with more traditional DMARDs such as methotrexate (63%) or sulfasalazine (55%). |
| Synthetic and biologic DMARDs | Kvien et al (133) | 2005 | - NOR-DMARD, a Norwegian 5-center register, was established in December 2000. - All DMARD prescriptions to patients with inflammatory arthropathies are included, and patients are followed longitudinally with a variety of assessments. - Methotrexate is the most commonly used DMARD in rheumatoid arthritis. - The percentage of patients who have received anti-TNF drugs in RA has been 22.5%. - The percentage of patients receiving anti-TNF drugs is considerably higher in 2004 than earlier, and criteria for prescribing anti-TNF drugs appear to be trending toward patients with less severe and active disease. |
| Synthetic DMARDs and glucocorticoids | Edwards et al (134) | 2005 | - Aim: describe use of DMARDs in the treatment of RA and changing trends in their use. - General Practice Research Database (GPRD) was used to describe DMARD use by patients with RA identified using ICD-9 codes. - Subjects were studied between 1987 and 2002. The prevalence and duration of individual DMARD use and changing trends in DMARD use were investigated. - Thirty-four thousand three hundred and sixty-four patients with RA were identified. Only 17,115 (50%) individuals were prescribed at least one DMARD during the study period. - The most commonly prescribed DMARD over the study period was sulphasalazine (46.3%) and then methotrexate (31.4%). - Use of methotrexate increased 17-fold (1.8% of all DMARD prescriptions in 1988 to 30% in 2002) whereas use of gold had fallen (13.2% to 2.3%). - Prednisolone was used in up to 50% of RA patients in any one year and remained fairly constant throughout the study period. |
